# Thermally Drawn Shape and Stiffness Programmable Fibers for Medical Devices

**DOI:** 10.1002/adhm.202403235

**Published:** 2024-12-31

**Authors:** Jiwoo Choi, Qindong Zheng, Mohamed E. M. K. Abdelaziz, Thomas Dysli, Daniel Bautista‐Salinas, Andreas Leber, Shan Jiang, Jianan Zhang, Ali Anil Demircali, Jinshi Zhao, Yue Liu, Nick W. F. Linton, Fabien Sorin, Xiaoting Jia, Eric M. Yeatman, Guang‐Zhong Yang, Burak Temelkuran

**Affiliations:** ^1^ Department of Metabolism, Digestion, and Reproduction, Faculty of Medicine Imperial College London London SW7 2AZ UK; ^2^ The Hamlyn Center, Institution of Global Health Innovation Imperial College London London SW7 2AZ UK; ^3^ Department of Bioengineering, Faculty of Engineering Imperial College London London SW7 2AZ UK; ^4^ National Heart and Lung Institute, Faculty of Medicine Imperial College London London SW3 6LY UK; ^5^ Institute of Materials École Polytechnique Fédérale de Lausanne Lausanne 1015 Switzerland; ^6^ Bradley Department of Electrical and Computer Engineering Virginia Polytechnic Institute and State University Blacksburg VA 24060 USA; ^7^ Imperial College Healthcare NHS Trust London W12 0HS UK; ^8^ Department of Electrical and Electronic Engineering Imperial College London London SW7 2AZ UK; ^9^ Institute of Medical Robots Shanghai Jiao Tong University Shanghai 200240 China; ^10^ The Rosalind Franklin Institute Didcot OX11 0QS UK

**Keywords:** medical application, multimaterial fiber, programmable fiber, shape memory polymer, thermal drawing

## Abstract

Despite the significant advantages of Shape Memory Polymers (SMPs), material processing and production challenges have limited their applications. Recent advances in fiber manufacturing offer a novel approach to processing polymers, broadening the functions of fibers beyond optical applications. In this study, a thermal drawing technique for SMPs to fabricate Shape Memory Polymer Fibers (SMPFs) tailored for medical applications, featuring programmable stiffness and shape control is developed. Rheological and differential scanning calorimetry analyses are conducted to assess SMP's compatibility with the proposed thermal drawing process and applications, leading to the production of multilumen, multimaterial SMPFs activated at body temperature. Different properties of SMPFs are investigated in three medical devices: stiffness‐adjustable catheters, softening neural interface, and shape‐programmable cochlear implants. Comprehensive characterization of these devices demonstrates the potential of thermally drawn SMPs to be employed in a wide range of applications demanding programmable mechanical properties.

## Graphical abstract

Thermal drawing technique is adapted to fabricate Shape Memory Polymer Fibers (SMPFs) with programmable stiffness and shape. Multilumen and multimaterial SMPFs are developed for innovative medical devices: stiffness adjustable catheters, body temperature softening neural interfaces, and shape programmable cochlear implants.

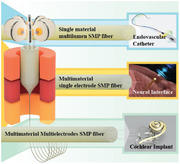

## 1 Introduction

Thermo‐responsive Shape Memory Polymers (SMPs) are a unique class of materials capable of retaining temporary shapes, which revert to their original form when exposed to external stimuli.^[^ ^1^ ^]^ This ability, known as the Shape Memory Effect (SME),^[^ ^1^, ^2^ ^]^ is triggered when SMPs reach their glass transition temperature (*T*_g_), causing a transition to a soft rubbery state. This state provides two important features: material softening and shape recovery.^[^ ^3^ ^]^ SMPs can present a broad *T*_g_ range from −70 to +100 °C, showing their wide availability to heat‐based stimuli requiring applications. They can also withstand high strains, up to 800%, which significantly surpasses the 7–8% maximum strain of Shape Memory Alloys (SMAs),^[^ ^3^ ^]^ another widely used stimuli‐responsive material in the medical field. A broad range of SMPs has been investigated for the development of various clinical concepts,^[^ ^4^ ^]^ including vascular plugs,^[^ ^5^ ^]^ self‐tightening sutures,^[^ ^6^ ^]^ thrombectomy devices,^[^ ^7^ ^]^ scaffolds,^[^ ^8^ ^]^ and catheters.^[^ ^9^ ^]^ However, the advancement of these SMP‐based devices to early‐stage human clinical trials remains notably scarce (e.g., TrelliX Embolic Coil System^[^ ^10^ ^]^ and VenoStent's Selfwrap^[^ ^11^ ^]^).

Thermal drawing is a promising fiber manufacturing technique with significant potential to expand the advantages of SMPs. This approach provides the ability of continuous fabrication of km‐long fibers with complex cross‐sections and the integration of diverse materials.^[^ ^12^ ^]^ Since its introduction,^[^ ^13^ ^]^ the multimaterial thermal drawing technique has been employed in various applications, such as fiber‐robots,^[^ ^14^ ^]^ artificial muscles,^[^ ^15^ ^]^ catheters,^[^ ^16^ ^]^ and neural interfaces/therapy‐monitoring implants.^[^ ^14^, ^17^ ^]^ Furthermore, exploring different constituent materials has resulted in fibers with various structural properties, such as flexibility and stretchability,^[^ ^18^ ^]^ which provide additional functions that are valuable for medical applications. Thus, advanced functional fibers with programmable stiffnesses and shapes can be achieved by adapting SMPs in the thermal drawing technique. Polylactic Acid (PLA), an SMP material that is unsuitable for fiber drawing, has previously been incorporated into polymeric fibers by encapsulation in an amorphous cladding to create shape‐recoverable fibers. However, this approach faced manufacturing limitations and safety concerns in medical applications due to high actuation temperature (>57 °C).^[^ ^19^ ^]^

In this study, we present an advanced set of shape and stiffness programmable materials we refer to as Shape Memory Polymer Fibers (SMPFs), which are thermally drawn Fibers from biocompatible SMPs, and explore their potential for healthcare use. We start our investigations by evaluating the compatibility of the commercial polyurethane‐based SMP (PUSMP) with the thermal drawing process using Rheological analysis and Differential Scanning Calorimetry (DSC) measurements. These measurements determine the temperature range required for thermal drawing and SMP actuation. Building on these analyses, multilumen, multimaterial programmable SMPFs were successfully fabricated using a custom‐built draw tower.

The proposed manufacturing approach facilitates the introduction of lumens and metal microwires into the fibers during the drawing process, allowing fluidic, electrical, and mechanical functions. Three variations of SMPFs were designed for three targeted applications carefully chosen to demonstrate the benefits of their disparate unique properties.

We first explore stiffness‐adjustable multilumen SMPFs designed to be used as endovascular catheters. In endovascular interventions, flexible and maneuverable catheters^[^ ^20^ ^]^ navigate through tortuous vasculature with minimal catheter‐tissue interaction, reducing the risk of vascular trauma and postoperative complications.^[^ ^21^ ^]^ However, catheters also need to be rigid and durable enough to withstand force application while preventing dislodgement from the target site. Commercially available catheters exhibit limitations in clinical settings, largely due to their fixed stiffness. Experimental variable‐stiffness catheters^[^ ^23^ ^]^ have been introduced to address this limitation, but they still have fabrication challenges^[^ ^22^, ^24^ ^]^ or safety concerns due to actuation temperatures exceeding the safe limit of 41 °C for endovascular interventions.^[^ ^23^, ^25^ ^]^ The stiffness adjustability of the multilumen SMPFs was investigated to assess whether they can be safely employed to address these issues. An SMPF‐based catheter was then developed with the necessary range of pliability, and its functionality was demonstrated in a phantom model.

The thermal drawing approach was then adapted to fabricate SMPFs with electrical communication designed to be used as an atraumatic neural interface. Previous neural interfacing applications have used passive polymer materials^[^ ^26^ ^]^ like polycarbonate (PC),^[^ ^27^ ^]^ polymethyl methacrylate (PMMA),^[^ ^28^ ^]^ polyethyleneimine (PEI),^[^ ^17b^ ^]^ or hydrogel^[^ ^29^ ^]^ due to their biocompatibility and low or adjustable mechanical stiffness. The mechanical mismatch between neural tissue and the fiber needed to be further reduced to minimize implant‐related trauma^[^ ^26^, ^30^ ^]^ and the associated chronic foreign body inflammatory response.^[^ ^26^, ^30^ ^]^ SMP‐based neural interfaces have also been previously studied to maximize the benefit of adjustable stiffness^[^ ^17d^ ^]^ or SME.^[^ ^31^ ^]^ However, the required *T*_g_ was higher than the body temperature, and a complex fabrication process was followed. Neural interfaces based on our thermally drawn multimaterial SMPFs have the potential to address these challenges, as the SMP allows the implant to soften dynamically at body temperature, thereby reducing force transfer into the surrounding neural tissue.

Finally, we investigated the shape recovery properties of multimaterial SMPFs to assess their potential use in Cochlear Implants (CIs). Implantation surgery often results in the iatrogenic loss of residual hearing due to abrasive damage to the delicate inner hair cells during electrode array insertion.^[^ ^32^ ^]^ Some attempts have been made to utilize SMPs and SMAs to resolve these issues. Nevertheless, both of these approaches did not integrate wires, neglecting one of the biggest challenges of CI fabrication,^[^ ^33^ ^]^ and the SMP approach demonstrated limited shape recovery ratio.^[^ ^34^ ^]^ CIs face technical and practical fabrication challenges,^[^ ^35^ ^]^ including predominantly manual assembly,^[^ ^36^ ^]^ increasing the per‐implant cost.^[^ ^37^ ^]^ Thermal drawing technology offers the potential to mass‐produce SMPFs with embedded multielectrode arrays. At the same time, SMPF's programmable SME could assist with personalized electrode placement and reduce iatrogenic harm during implantation.^[^ ^38^ ^]^

These SMPFs showcase the synergy created by combining the unique properties of SMPs with the benefits of the thermal drawing approach through the demonstrated medical devices.

## 2 Results and Discussion

### 2.1 Material Characterization of SMP

**Figure 1:**
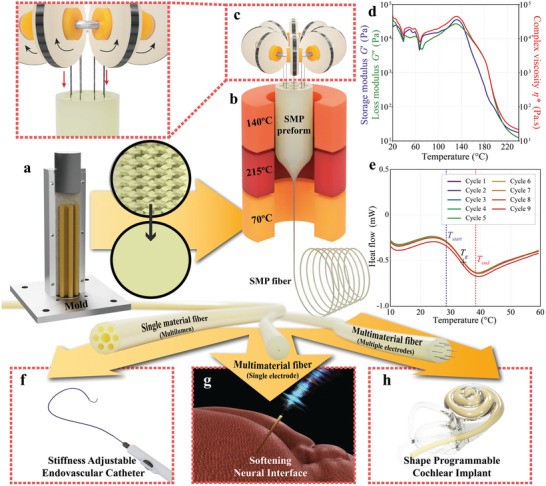
Development of the thermal drawing process of SMPFs and proposed medical applications. a) A custom mold with multiple pillars. b) 3‐zone thermal drawing platform. c) A custom wire feeding platform. d) The oscillatory shear rheological analysis results for MM3520, showing storage modulus, loss modulus, and complex viscosity as a function of temperature. e) DSC analysis result of MM3520 in 9 heating and cooling cycles f) SMP Endovascular Catheter. g) SMP Neural Interface. h) SMP CI.

A biocompatible^[^ ^39^ ^]^ and commercially available PUSMP, MM3520, was chosen for the thermal drawing as its nominal *T*_g_ range (35 °C) is well suited to use human body heat as an external stimulus. PUSMP's suitability for use in our thermal drawing process, illustrated in **Figure** **1**a–c, was determined by performing rheological measurements. This evaluation, based on previous rheological analysis for fiber drawings,^[^ ^18^, ^40^ ^]^ is critical as the polymer must exhibit a sufficiently high viscosity at the drawing temperature to prevent breakage while also allowing continuous flow during the drawing process. Analysis of viscoelastic properties, specifically the storage modulus (*Gʹ*) and loss modulus (*Gʺ*), across temperatures, is important to determine an appropriate temperature range for fiber drawing. *Gʹ* describes the material's ability to preserve structural integrity, while a stable *Gʺ* indicates resistance to applied stress. The result shows a quick decline in the *Gʹ*, contrasted with a more gradual reduction in the *Gʺ*, leading to a noticeable crossover point. Based on these rheological analyses (Figure 1d), we determined that the temperature window of the SMPF drawing process has a lower limit at 147 °C, at which point the complex viscosity (*η*) remains sufficiently high (≈10^4^–10^2^ Pa.s). These analyses reveal a narrow temperature range within which the material can be thermally drawn and are also comparable to common thermal drawing materials like PC or Styrene‐Ethylene‐Butadiene‐Styrene (SEBS).^[^ ^14^ ^]^ In addition, the *T*_g_ value was calculated (using the rheological measurements) as 32.2 ± 1.3 °C, enabling us to define two crucial temperature regions: rubbery state (*T*_g_ ≤ *t*, *t* = operation temperature) and glassy state (*T*_g_ > *t*).

In addition, the furnace temperature of the draw tower was set to 215 °C. This set furnace temperature differs from the actual temperature of the preform during the drawing. This difference is specific to each system as it depends on the furnace size and heating system and can reach up to 60 °C. This difference has been investigated before for a draw tower with the same identical furnace.^[^ ^28^, ^41^ ^]^ There are multiple factors why the furnace temperature setting is higher than the draw temperature. The draw tower furnace has a replaceable glass tube between the preform and the heating elements, which helps protect the furnace. Also, the top and bottom of the furnace are open, which contribute to the heat loss. In our measurement, when the temperature monitor of the middle zone was stabilized at the draw temperature of 215.0 ± 0.2 °C (measured by the thermocouple of the furnace), the preform surface was at 157 ± 2 °C (measured by a thermocouple attached to the preform outer surface). Thus, the drawing starts at a temperature slightly above the suggested minimum of the temperature window (147 °C).

DSC was performed to evaluate the consistency and reliability of the PUSMP under thermal stress after multiple heating and cooling cycles, as shown in Figure 1e and Figure S6 (Supporting Information). The *T*_g_ was calculated at 34.1 ± 0.6 °C using the tangent intersection method. This result is consistent with the *T*_g_ value obtained from our earlier rheological analysis. The small discrepancy in *T*_g_ between these methods (2 ± 1 °C) may arise because DSC identifies *T*_g_ based on changes in heat capacity, whereas rheology detects *T*_g_ from changes in viscosity. Thus, the consistency in DSC analysis results indicates that repeated heating and cooling cycles are not expected to affect the performance of the SMP material.

The average onset temperature (*T*_start_) and endset temperature (*T*_end_) were also measured from the DSC analysis result. *T*_start_ represents the beginning of significant heat flow changes (indicating softening of materials), while *T*_end_ shows the completion of these changes at above *T*_g_. This measurement suggests that the temperature should be reduced below 27.1 ± 0.5 °C (*T*_start_) to maintain a glassy state. A significant shape recovery and softening can be expected when the material is exposed to a body temperature that is close to 38.7 ± 0.7 °C (*T*_end_).

Based on these measurements, we explored the benefits of SMP and thermal drawing in a medical context by characterizing different designs of SMPFs and adapting them to medical devices: a catheter with adjustable stiffness, a neural interface softening at body temperature, and a shape‐recovering CI.

### 2.2 Endovascular Catheter: Stiffness Control of Multilumen SMPF

**Figure 2:**
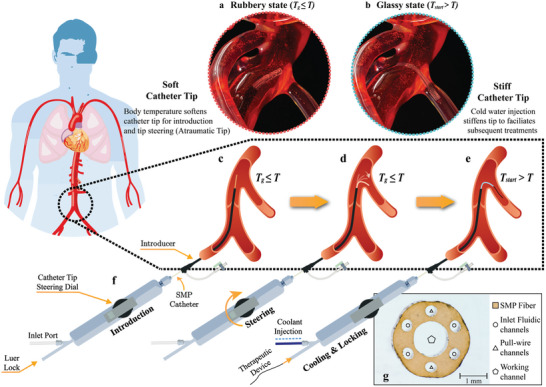
Overview of stiffness‐adjustable steerable catheter design and actuation. a,b) Schematics of the catheter tip in its soft (a) and stiff (b) states. c) The SMP catheter is introduced into the vessel and advanced to reach a specific branch. As body temperature (*T*) is above the material's *T*_g_, the catheter is initially soft and flexible. d) At the vessel's branching point, the surgeon can deflect the catheter's distal end using a conventional pull‐wire actuation mechanism. e) Once the surgeon is satisfied with the catheter's position, a coolant infusion reduces the catheter temperature below *T*_start_, causing the catheter to become stiff. This increased rigidity makes the catheter significantly more stable and harder to dislodge from the target position. f) A handle was fabricated using a 3D printer (Object500 Connex3, Stratasys Ltd., USA) to facilitate manual catheter control. g) A microscope view of the catheter's cross‐section (VHX‐2000, Keyence Corporation, Japan). The adult male image is acquired from Depositphotos, Inc., USA.^[^ ^42^ ^]^

The dual demands on catheter stiffness, illustrated in **Figure** **2**a–e, are addressed by incorporating multilumen SMPFs into a steerable catheter. The highly scalable thermal drawing technique is used to overcome the difficulties of fabricating long, inexpensive SMPFs with complex cross‐sections. In order to fulfill the diverse functions, the cross‐section of the catheter (Figure 2g) was designed and conceptually optimized by a series of thermal simulations in Figure S1 (Supporting Information). The resulting SMPFs (2.4–2.8 mm diameter) featured six side lumens (0.4 mm diameter) and one central lumen (1.3 mm diameter).

**Figure 3:**
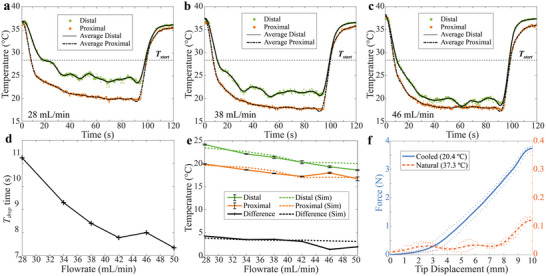
Characterization of stiffness adjustability. a–c) Mean temperature change at the proximal and distal ends of the catheter with coolant injection at a) 28, b) 38, and c) 46 mL min^−1^. d) Temperature drop (*T*_drop_) time at the distal end using different coolant flow rates. e) Minimum temperatures at the proximal and distal ends at different flow rates and the subsequent temperature differential along the catheter's length. The solid and dashed lines represent experimental and simulation results, respectively. f) Force measurements of stiff (cooled stiff state, 20.4 ± 0.2 °C) and soft (natural soft state, 37.3 ± 0.1 °C) SMPF catheters during the flexural rigidity tests with the coolant injection at 38 mL min^−1^. Mean ± SD, *n* = 3.

Overall, device temperature can be controlled by adjusting coolant injection against passive heating from body temperature, enabling on‐the‐fly stiffness characterization. The cooling system supplied up to 50 mL min^−1^ into the catheter's fluid lumens. This upper limit is safely below the typical catheter‐based intravascular flow injection operations, such as contrast injection (1.5–5 mL s^−1^).^[^ ^43^ ^]^ **Figure** **3**a–c demonstrates the temperature changes observed at the catheter's proximal and distal ends during coolant injection with various flow rates. The coolant (water) was chilled to 4.0 ± 0.1 °C using a refrigerated circulator (RHC‐800, Cole‐Parmer Instrument Company, LLC, USA). While water was used as the coolant in this study, 0.9% saline solution can be used in clinical practice, which is a standard cleaning or cooling fluid in various medical procedures. This is similar to the standard cold saline infusion for inducing hypothermia, in which 4 °C saline can be administered at a rate of 100 mL min^−1^, with a total volume of up to 2100 mL (volume for an average 70 kg patient, with the suggested amount of 30 mL kg^−1^).^[^ ^44^ ^]^ Such operation is usually proceeded peripherally, meaning that minor vessels are involved.^[^ ^44^ ^]^ Considering the size of our catheter, the vessels accessible are much larger than those and thereby more tolerable to coolant injection. Given a similar coolant temperature and larger operation vessel diameter, we can conclude that our approach of stiffening SMP via injecting coolant is safe for patients. A summary of relevant intravascular flow injection operations is included in Table S1 (Supporting Information).

After coolant injection, temperatures at both the proximal and distal ends dropped and stabilized after several seconds. The SMP rubbery‐glassy transition response time, representing the time needed to cool the catheter's distal end from ambient body temperature (i.e., 37 °C) to *T*_start_, was measured in the range of 7–11 s (Figure 3d). This cooling time is reasonable considering 1) the duration of endovascular interventions, which usually exceeds 20 min,^[^ ^45^ ^]^ and 2) any additional time required to reposition the catheter in case of dislodgement.^[^ ^46^ ^]^ The recorded temperatures were consistently lower than *T*_start_, with the lowest measured values of 16.7 ± 0.5 and 18.4 ± 0.2 °C at the proximal and distal ends, respectively (Figure 3e). The measurement results closely align with the outcomes predicted by a Finite Element Analysis (FEA) simulation.

Flexural rigidity measurement was performed to assess the stiffness of the SMPF without coolant injection (natural soft state, 37.3 ± 0.1 °C) and with 38 mL min^−1^ coolant injection (cooled stiff state, 20.4 ± 0.2 °C measured at the distal end). This coolant flow rate was selected to achieve a distal end temperature below *T*_start_ while minimizing coolant injection volume. Considering a safe saline infusion volume of 30 mL kg^−1^ for adults,^[^ ^47^ ^]^ our catheter operating at this flow rate enables maintaining a stable and rigid state for over 30 min. Also, it balances patient safety with an adequate operational duration to maintain the stiff state, considering the total volume injected in similar procedures, as shown in Table suppinfo1 (Supporting Information).

The SMPF exhibited a 31‐fold increase in stiffness from 12.6 ± 0.5 to 374.5 ± 9.1 mN mm^−1^ (Figure 3f). The flexural rigidities calculated from this measurement are 5.3 ± 0.2 and 156.0 ± 3.9 Nmm^2^, respectively. The flexural rigidity in the stiff state exceeds that of commercial catheter sheaths that are on the stiffer side of the spectrum, such as Splitsheath from Optimed, Germany (2.9 mm OD, 130.0 ± 2.4 Nmm^2^), as depicted in Figure S2 (Supporting Information). Additionally, while in its soft state, SMPF's flexural rigidity is below the values measured from softer products like a custom inner tube from Ecomedis, Germany (1.5 mm OD, 28.4 ± 0.1 Nmm^2^). These flexural rigidity values of SMPF in both states are also comparable to the previously reported values of commercial catheters (e.g., AXS Infinity (Stryker Corporation, USA),^[^ ^48^ ^]^ and Launcher (Medtronic plc, Ireland).^[^ ^16b^ ^]^ Thus, SMPF‐based catheters can achieve both the flexibility needed for navigation and the stability required to prevent accidental dislodging from the target position.

The SMPF‐based catheter's achievable duel stiffness is comparable to previously introduced variable stiffness catheters.^[^ ^9^, ^23^, ^49^ ^]^ Our catheter offers a competitive cooling time and stiffness adjustability change within a safe temperature window for clinical scenarios, as shown in Table S2 (Supporting Information). While the magnetic stiffness adjustable catheter, for example, needed over 60 °C to reach a soft state^[^ ^9^, ^23^ ^]^ or 107 s cooling time to achieve a stiff state,^[^ ^9^ ^]^ the SMPF‐based catheter showed over 31‐fold stiffness change in 8.4 s within a safe temperature range of 20–37 °C. In addition, rather than stiffness control of short segments^[^ ^49^ ^]^ or the steering tip,^[^ ^23d^ ^]^ our approach enables stiffness control over the whole length of the catheter. These advantages allow more stable shape locking during interventions.

**Figure 4:**
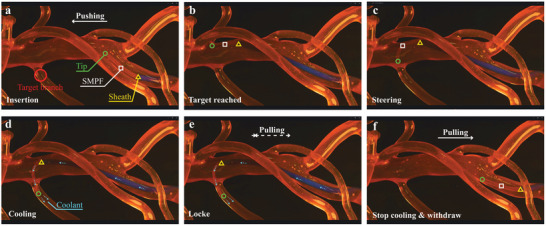
Sequential steps in the phantom study procedure. a,b) SMPF catheter, in its soft state at body temperature, is introduced into the vessel and advanced to reach the target branch. c) The catheter's distal end is deflected toward the target branch using a conventional pull‐wire actuation mechanism. d) Coolant is injected to switch the catheter into its stiff state. e) When a pulling force is applied at the proximal end, the catheter retains its shape and does not dislocate from the target branch. f) Cessation of coolant flow enables passive heating from body temperature, hence returning the SMPF catheter to its soft state for easy removal.

A phantom study was performed to showcase the feasibility of this SMPF catheter in clinical scenarios. Nitinol tendons were integrated into multilumen SMPF fiber, and it was assembled in a 3D‐printed handle to facilitate manual control, resulting in a steerable catheter with adjustable stiffness (Figure S3, Supporting Information). The catheter shaft was encased in a sheath (blue) to mitigate accidental bending of the shaft induced by the tension from the tendons. The catheter was inserted through an introducer into the right femoral artery and then advanced along the major pathway of the artery to reach the targeted vessel branch (**Figure** **4**a,b). The catheter was initially in a soft state (circulating water temperature in the phantom is 37.0 ± 0.4 °C) to facilitate easier endoluminal navigation and minimize the risk of vessel wall trauma. At the vessel's branching point, the catheter tip was then maneuvered and guided into the target branch (Figure 4c). Once satisfied with the catheter's position, a coolant (4.0 ± 0.1 °C) infusion was initiated to reduce the catheter temperature below *T*_start_, hence causing the material to transition into its stiff state. (Figure 4d). When a pulling force was applied at the proximal end, the catheter retained its locked U‐shape and did not dislocate from the target branch. (Figure 4e). In contrast, stopping the coolant injection allowed the circulating water to passively increase the catheter temperature above *T*_g_, causing the material to transition into its soft state and be effortlessly withdrawn from the vessel. This phantom study (Video S1, Supporting Information) showcases the entire procedure for using stiffness‐adjustable catheters in endovascular navigation. Stiffened SMPF locks the shape of the catheter, preventing any dislocation from the target. At the same time, the softened SMPF provides sufficient flexibility for safe and agile navigation.

### 2.3 Neural Interface: Body Temperature Softening Multimaterial SMPF

This study investigates the potential of SMPFs for implementation as needle‐like neural interfaces, in which electrode functionality is provided by an integrated microwire (Figure 1f). The thermal drawing process enables seamless integration of conducting wires into fibers.^[^ ^14a^ ^]^

The fiber was drawn at 0.3 mm diameter to evaluate the softening we can achieve within the diameter range of commonly used needle‐like neural interfaces.^[^ ^27^, ^29^ ^]^ The diameter can be reduced further to lower the stiffness, which improves the biocompatibility.^[^ ^50^ ^]^ However, reducing the diameter will also increase fragility, which is a critical consideration for safe insertion. We successfully incorporated 0.03 mm Platinium/Irridium (Pt/Ir) wire as the recording electrode in the center in a 0.3 mm diameter SMPF (*T*_g_ = 35) using the thermal fiber drawing method to minimize any post‐draw processes and enhance biocompatibility.

**Figure 5:**
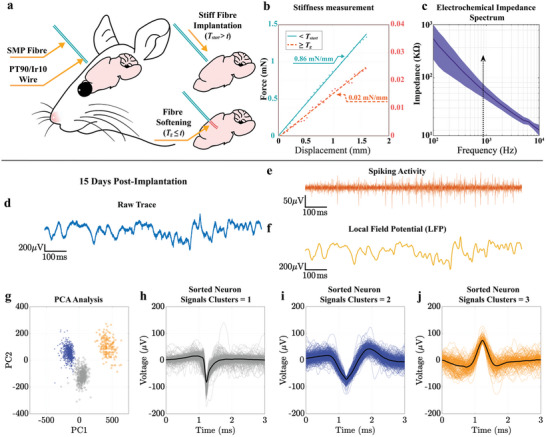
SMPF‐based neural interface's implantation process, characterization, and measurement during the 15 days post‐implantation. a) Implantation process. b) Stiffness measurement of neural interface. Mean ± SD, *n* = 3. c) Electrochemical Impedance Spectrum. *N* = 5 d) Raw neural signal (unfiltered). Solid line and shaded areas in the figure represent mean and standard deviation, respectively. e) High‐frequency bandpass‐filtered signal (300–5000 Hz, capturing single‐unit activities). f) Low‐frequency bandpass‐filtered signal (0.3–300 Hz, representing Local Field Potential (LFP). g) Principal Component Analysis (PCA) of neural signals. h–j) Clustered neural signals.

In the neural interface implantation procedure, the device rigidity at room temperature prior to implantation surgery aids the implantation. As the body temperature is above *T*_start_, the stiffness of the SMPF‐based neural interface transforms upon contact with brain tissue, enabling the softened neural interface to accommodate complex biological environments with improved biocompatibility (**Figure** **5**a). To evaluate the suitability of SMPFs for neurological implantation, we characterized their stiffness under two relevant temperature conditions (Figure 5b). The SMPF exhibits a stiffness of 0.86 mN mm^−1^ at 25 ± 1 °C (room temperature) and 0.02 mN mm^−1^ when it is exposed to heat at 37.0 ± 0.5 °C (over *T*_g_, namely body temperature). This result shows that, even with a slightly lower temperature from *T*_start_ (27.1 °C), a 43‐fold increase in stiffness is achievable.

This atraumatic neural implant is also comparable to previously reported approaches using materials with low Young's modulus. Previous studies surrounded stiff materials (e.g., Cyclic Olefin Copolymer(COC), Polysulfone(PSU), or PC) with relatively soft or softening claddings (e.g., Fluorinated Ethylene Propylene(FEP), hydrogel) to minimize the damage to brain cells they contact in long‐term implementation.^[^ ^27^, ^29^ ^]^ However, these approaches still rely on stiff core materials, and the Young's moduli of these probes are still higher than the Young's modulus of our SMPF‐based neural interface. For instance, the hydrogel‐based neural interface, another dual stiffness approach, showed a 6‐fold reduction in Young's modulus from 2.19 to 0.35 GPa,^[^ ^29^ ^]^ while the PC/FEP‐based neural interface was fixed at 0.53 GPa.^[^ ^27b^ ^]^ The SMPF‐based neural interface presents a decrease in Young's modulus from 0.72 to 0.02 GPa, more than a 36‐fold difference, which is sufficient for both brain insertion and long‐term implantation, providing improved biocompatibility.

The electrical performance of the electrodes was characterized prior to surgical implantation (Figure 5c) by electrochemical impedance spectroscopy with an average impedance value of 70.6 kΩ at 1 kHz. After conducting the impedance measurements, the SMPF‐based neural interface was implanted into the hippocampus of wild‐type mice brains (*n* = 5) to evaluate its performance in the accurate identification of neuronal activity. Figure 5d–f shows the measurements after 15 days post‐implantation. From the spiking activities, three well‐isolated spike clusters can be differentiated using PCA and K‐means clustering (Figure 5g–j). A greater degree of spike‐cluster separation is useful as it proves the accuracy and reliability of detecting and classifying neural signals.^[^ ^51^ ^]^ Two commonly used metrics, L‐ratio and Isolation Distance (ID), were calculated to evaluate spike‐cluster separation. L‐ratio is used to quantify how well a spike cluster is separated from noise, and other clusters with lower values indicate better separation.^[^ ^52^ ^]^ ID measures the distance from a spike cluster's center to the nearest point that would double the cluster's size, with higher values indicating better cluster isolation.^[^ ^53^ ^]^ The resulting L‐ratio and ID of each two clusters from those three clusters were 1.4 × 10^−4^ and 68.5, 3.4 × 10^−4^ and 49.1, and 5.8 × 10^−11^ and 283.5, respectively. These results demonstrate high‐quality spike sorting, as indicated by the low L‐ratio values and high IDs, which reflect clear separation and robust isolation of the clusters. This confirmed that the neural recordings using the SMPF‐based neural interface accurately identified neuronal activity. Besides the 15 day recording results shown in Figure 5d–j, we have assessed the chronic neural recording capability of our SMP‐based neural probe 35 days post‐implantation and observed one well‐sorted neural signal from the filtered spiking data (Figure S4, Supporting Information). We believe that the SMP‐based neural probe also has the potential to track individual neurons in long‐term periods.

In this study, the well‐isolated neural activities of the recorded signal further demonstrate the stability and potential in long‐term recording capability of the SMPF‐based neural interface with single‐unit resolution. By employing thermal drawing technology, SMP and electrodes are also combined during the drawing process, simplifying the fabrication process of neural interfaces.^[^ ^31^, ^54^ ^]^ The softening SMPF will aid insertion by preventing buckling and minimizing any potential post‐implantation damage to brain cells, offering enhanced opportunities for neural recording with reduced manufacturing cost and design flexibility.

### 2.4 Cochlear Implant: Shape programmable Multimaterial SMPF

CI implantation surgery often results in the iatrogenic loss of residual hearing due to abrasive damage to the delicate inner hair cells during electrode array insertion. An atraumatic shape programmable SMPF‐based CI was developed (Figure 1g), building upon our multimaterial fiber drawing approach.^[^ ^34^ ^]^ This fabrication method offers the potential to produce kilometers of cost‐effective, functionally rich fibers with multiple microwires at minimal diameters, overcoming traditional CI fabrication challenges. A circular array of eight stainless steel electrodes (0.03 mm in diameter) was positioned within the SMP preform to be integrated into the fiber during the drawing process. We produced SMPFs with 0.6 mm (± 0.05) and 1 mm (± 0.05) diameters having 8 electrode arrays. The thermal drawing approach facilitates the precise and controlled integration of multiple electrodes into a compact form, thereby enabling the fabrication of miniature implants at kilometer‐length scales.

**Figure 6:**
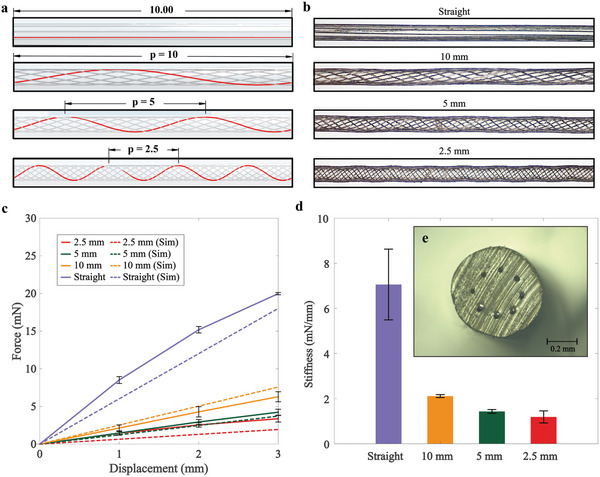
Characterization of SMPF with straight and helical wires. a) Illustration of SMPFs with straight wires and helical wires with three different pitches. b) Microscopic lateral views of SMPFs with eight straight and helical wires. c) Simulation and empirical analysis of force and deflection in SMPFs with straight and helical wires. d) Stiffness measurement of SMPFs. e) Microscopic image of SMPF's cross‐section. Mean ± SD, *n* = 3.

Increasing the number of electrodes enables a greater recovered hearing bandwidth; however, this also results in stiffer and more traumatic SMPF‐based implants with reduced shape recovery. This effect is exacerbated by the off‐center placement of wires when the fiber is bent, causing extra tension on the outside of the central axis and compression on the inside (Figure S5, Supporting Information). We propose that helically routed wires within the SMPF (**Figure** **6**a) distribute the tensile and compressive loads over shorter segments of each wire, potentially resulting in an improved shape recovery rate.

FEA simulation in COMSOL Multiphysics (Comsol AB, Sweden) was conducted to understand the change in stiffness on twisted SMPF compared to straight. Fibers with straight and helically arranged wires were then produced to validate these simulation results. Helical routing was achieved by twisting the drawn SMPFs around their longitudinal axis.

Figure 6b shows lateral microscopic images (Axio Zoom.V16, Carl Zeiss AG, Germany) obtained to confirm the arrangement of straight wires (0 rotations/mm) and helical wires with pitches of 10 mm (0.1 rotations/mm), 5 mm (0.2 rotations/mm), and 2.5 mm (0.4 rotations/mm) within the SMPF. The helical axis was observed to deviate slightly from the central axis of the fiber, with this deviation becoming more pronounced as the pitch length decreased; however, the wires successfully retained a consistent helical pitch.

Figure 6c presents a comparison between the manually measured bending forces and the FEA‐simulated bending forces at the distal tips of 1.0 ± 0.05 mm diameter SMPFs with straight and helical wires. Figure 6d shows the corresponding stiffness change. The results indicate that SMPFs with helical wires exhibit lower bending force than SMPFs with straight wires, reduced by over 86.2%. Minor overestimations observed in the simulation results could be related to the slight irregularities in the fiber diameter, especially due to twisting, which may result in a large impact on its stiffness. Regardless, the decrease in stiffness with increased pitch is clearly observed in both simulation and experimental results. This indicates that this is an effective strategy to reduce stiffness, leading to an improved shape recovery.^[^ ^27b^ ^]^ The stiffness of this 6 mm outer diameter SMPF with 8 wires is similar to that of the 0.3 mm outer diameter SMPF used for the neural interface, which is thinner and has only a single Pt/Ir wire at the center (Figure 5b).

The cross‐sectional microscope image (VHX‐2000, Keyence Corporation, Japan) in Figure 6e shows the integrity of a 0.59 mm diameter fabricated SMPF with eight wires. This tight integration of wires proves that introducing SMPs in thermal drawing technology can significantly reduce the current labor requirement for the manual CI fabrication process.

**Figure 7:**
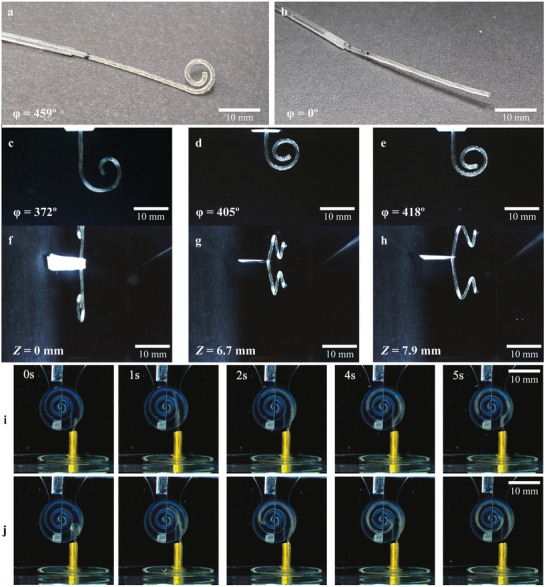
Shape recovery of SMPFs with straight and helical wires and demonstration using a three‐dimensional cochlear spiral phantom. The permeably programmed molding angle at 459°. a) An SMPF with helical wires after programming. b) A programmed SMPF with helical wires after straightening. c–e) Shape recovery of SMPFs with straight wires and helical wires with 5 and 2.5 mm pitch. f–h) Lateral view of SMPF after shape recovery. i) The phantom study with SMPF with straight wires. j) SMPF with helical wires in 10 mm pitch.

Their flexibility, rapid shape recovery, high electrode density, and low‐cost manufacturing scalability make helically wired SMPFs ideally suited for CI development. To fully evaluate this potential, we programmed the SMPFs’ permanent shape (**Figure** **7**a) by molding them within a phantom that imitated the cochlea anatomy. A molding temperature of 140.0 ± 6.0 °C (*T*_mold_) was used to set the SMP's programmed shape. Molding temperatures below *T*_mold_ resulted in inconsistent nonplanar deformation after molding due to the nature of twisted fibers. In contrast, molding temperatures above *T*_mold_ caused the SMPF to adhere to the mold and warp. Following the permeant shape programming, the SMPF was released from the mold while maintaining the straightened shape and cooled at room temperature (Figure 7b). This cooling transitioned the SMPF to a stiff state and effectively completed a simplified programming cycle. After this procedure, the changes in SME were analyzed by inserting straightened SMPFs into a water bath at 39.0 ± 0.2 °C (Figure 7c–e). All SMPFs completed shape recovery within 4 s. The shape recovery rate, which indicates the ability of a polymer to return to its original shape after exposure to a stimulus, is calculated by comparing the initial deformation angle (total spiral angle after molding) to the final angle after shape recovery. The shape recovery rate was also improved from 81.1% (straight) to 88.3% (pitch = 5 mm) and 91.1% (pitch = 2.5 mm), indicating that the previously demonstrated reduction of bending stiffness leads to a measurable improvement in shape recovery.

An additional shape change was observed from the lateral views in Figure 7f–h. While an SMPF with straight wires exhibited a planar recovery (*Z* = 0 mm), nonplanar deflections were observed for SMPFs with helical wires. This is because twisting introduces radial stress to the SMPF, causing it to bend out of a plane when recovered. Thus, the relationship between pitch and 3‐dimensional recovery allows for further programming of SMPFs to better align with the cochlear height of a specific patient, presenting a further opportunity to reduce the traumatic effects during insertion.

Implantation of the SMPF‐based CIs was then evaluated using a 1:1.3 scaled 3D printed phantom of the labyrinthine anatomy. The straightened 0.6 ± 0.05 mm diameter SMPFs were inserted through a cooled copper tube (10–15 °C, below *T*_start_) into the opening of the Scala Tympani (ST), representing the oval window. Images with 1‐s intervals during recovery SMPFs with straight and helical wires are shown in Figure 7f,g, respectively, and Video S2 (Supporting Information) demonstrates the SMPF‐based CI SME in the water bath and the phantom. Shape recovery begins at the distal tip and progresses toward the proximal end as the SMPF was gradually inserted further into the phantom heated at 39.0 ± 0.2 °C. The result shows that the shape recovery angle was improved from ≈210° (straight) to over 370° (pitch = 10 mm). This is an improvement from 46% to 82% of the original mold angle (459°), with improved insertion depth from 20 to 30 mm.

In clinical scenarios, our improved insertion depth of the SMPF‐based CI could improve sound quality and speech perception with stable placement.^[^ ^33b^ ^]^ Our result is comparable to the previously reported SMA‐based neural interface proposing to improve the insertion depth^[^ ^33b^ ^]^ but without the presence of electrodes.

## 3 Conclusion

This study explores the use of stiffness and shape programmable SMPFs for advanced healthcare applications. By combining the benefits of thermal drawing and SMPs, three different SMPF configurations were investigated. The stiffness adjustability of SMPFs was shown in a multilumen endovascular catheter and a multimaterial neural interface, while SME of SMPFs was explored for a multimaterial CI.

SMPFs with hollow lumens were customized for stiffness‐adjustable catheters. This enabled the catheter to navigate through tortuous anatomies at a lower stiffness and rigidify on demand, providing adequate support for manipulating equipment without dislodging from its target branch. This design proves value in clinical scenarios, such as positioning pacing leads into branches of the coronary veins or other vascular interventions, such as renal artery stent implantation. SMPF‐based catheters can also be helpful in defining a rigid pathway to advance other catheters and guidewires through tortuous bends of the vascular system, increasing the procedure's safety by reducing the forces applied to the vessels at the bends.

Our catheter design provides an adjustable stiffness range that matches various catheters and sheaths within a safe temperature range for patients. Given the advantages of thermal drawing, our design enables cooling along the entire catheter while retaining a competitive cooling time and stiffness variance compared to existing variable stiffness catheters.^[^ ^9^, ^23^, ^49^ ^]^ While the amount of coolant used for this unidirectional cooling approach is safe for patients, a further study on circulating cooling fluid may eliminate direct injection of coolant into the body, providing a longer and safer intervention period. In addition, the softening of the SMPFs can also be achieved by running a small current through embedded resistive wires that can be seamlessly introduced into the catheters.^[^ ^14^, ^23^ ^]^ These approaches will help expand the benefits of SMPF‐based catheters to natural orifice transluminal endoscopic interventions (e.g., colonoscopy, bronchoscopy, and ureteroscopy) to address patient discomfort and other adverse effects.

SMPFs can also facilitate electrical communication, serving as the softening neural interfaces via conducting wires fed into the fiber during the thermal drawing process. Beyond serving as the protective layer of the electrode as any other passive polymer, the SMPF was shown to be significantly softer at body temperature compared to its stiff state at room temperature, thereby reducing implant‐related trauma. The Pt/Ir electrode encapsulated inside the SMP layer enabled long‐term chronic recording, allowing us to investigate the underlying neural circuitry. Although we observed increased noise after 35 days, we could still sort one clear cluster, as shown in Figure S4 (Supporting Information). To enhance the implantation procedure and achieve longer stability in recording, further studies are needed focusing on improving the SMPF‐based neural interface's design, such as introducing a bevel‐cut at the fiber's tip to achieve large contact areas with neurons and optimizing fiber diameter. In an expanded animal study, probes with improved designs could then be compared with the state‐of‐the‐art in long‐term implantation, demonstrating their biocompatibility through an assessment of minimized post‐implantation damage to brain cells and their capacity for single‐neuron activity tracking. Moreover, building on the multifunctional nature of the thermal drawing platform, the number of electrodes could be increased to monitor multiple neuron activity, and microchannels could be introduced for drug delivery.^[^ ^14c^ ^]^ A softening SMPF implant approach can be adapted to design other medical devices, such as infusion needles or drainage catheters (e.g., external ventricular drains).

The potential of SMPFs as bespoke shape programmable CIs was also highlighted. Despite having a thicker outer diameter and additional wires when compared to the SMPF used in the neural interface, the stiffness of the SMPF‐based CI remained similar due to the helical routing of the wires. While the SMP enabled shape recovery in 2 dimensions, twisting the drawn fibers introduced a potential for nonplanar programmability in the 3rd dimension, providing a personalized fit within the cochlea.^[^ ^35b^ ^]^ This SMPF‐based CI has successfully demonstrated shape recovery at a near anatomically relevant scale. This SMPF‐based CI has successfully demonstrated shape recovery at the near anatomically relevant scale. The SMPF‐based CI design could improve patient outcomes by preserving residual hearing during insertion and improving the sound quality with the increased insertion depth,^[^ ^55^ ^]^ while the low manufacturing cost from this simplified mass production approach will widen access to CIs.

It's crucial to emphasize that additional research into expanding the number of conductive wires remains necessary. Contemporary CIs utilize between 12 and 22 electrodes to potentially enhance and optimize sound quality and speech recognition. Our study, however, was confined to using only 8 wires. Increasing this number could affect the drawability, stiffness, and shape recovery of SMP fibers, as well as influence the twisting pitch of the fiber to accommodate precise shape recovery programming. It is possible to address this limitation by reducing the diameter of the wires by employing metallic glasses. These materials can be softened and drawn together with SMP,^[^ ^56^ ^]^ allowing the integration of a higher number of electrodes with much smaller diameters. Thermal drawing could be adapted to achieve fibers with helical structures by spinning the preform during the drawing process.^[^ ^16^ ^]^ This modification will produce SMPFs with helically routed electrodes during the draw, eliminating the post‐draw twisting step. In addition, the SMPF‐based CI could be combined with robotic insertion, resulting in more precise control of its shape recovery to increase insertion depth can be expected.^[^ ^17^ ^]^ Hence, further research could be conducted on person‐to‐person customization to the unique individual cochlear shapes through the fiber twisting process, increased number of electrodes, and robotic insertion.

SMPFs in this study were fabricated using a biocompatible PUSMP^[^ ^39^, ^57^ ^]^ with robust stability under physiological conditions, making it suitable for long‐term implantable medical devices. Moreover, a closely related PUSMP from the same manufacturer showed excellent cytocompatibility with fibroblasts, no cytotoxicity, and reduced platelet adhesion when compared to commercial medical grade PU in their 7‐day in vitro result,^[^ ^58^ ^]^ further evidencing its suitability for medical devices. In addition, using other types of PUSMPs as a filler for intracranial aneurysms showed minimal inflammation, significant endothelialization, and neointima formation over 90 days of in vivo study^[^ ^59^ ^]^ and the ability to maintain mechanical integrity and shape recovery over two years.^[^ ^60^ ^]^ These studies present strong evidence for the potential long‐term biocompatibility and stability of the SMPFs proposed in this work.

It is worth noting that PUSMP used in this study is also known as moisture‐responsive material, which becomes softer when exposed to moisture for a long time.^[^ ^39^, ^61^ ^]^ This additional softening might be beneficial for long or permanent implantation. A hydrogel‐based neural interface has already been used to demonstrate that moisture can be used as a stimulus to reduce stiffness.^[^ ^29^ ^]^ Thus, this softening mechanism will also be applicable to the SMPF‐based neural interface or CI. Nevertheless, absorbing moisture limits the functionality of the devices requiring stiffness adjustment.^[^ ^61^ ^]^ Additional hydrophobic coating or tubing can minimize any moisturization and potential degradation of the SMPFs. We anticipate our initial demonstrations, along with existing work on this material's biocompatibility, will motivate further research to investigate these potential issues and provide solutions.

In conclusion, thermally drawn SMPF is an innovative solution for the shape and stiffness programmable medical devices. The thermal drawing technique offers a scalable and cost‐effective promising pathway to integrate multiple materials together with SMPs and customize fiber properties. These SMPFs can address current clinical challenges while encouraging the development of other high‐aspect‐ratio devices for disparate healthcare applications and beyond.

## 4 Experimental Section

### Rheological analysis, DSC, and SMPF Fabrication—Material Preparation

Biocompatible^[^ ^39^ ^]^ commercially available PUSMP pellets (MM3520, SMP Technologies, Inc., Japan) were purchased. To reduce any moisture absorbed within the pellets, they were dried in a vacuum oven (Vacucell EVO 55, MMM Medcenter Einrichtungen GmbH, Germany) at 80 °C for a minimum of 12 h. While the specific ingredients of this material have not been disclosed, potential ingredients for this polymer can be found in the manufacturer's material safety data sheet.^[^ ^62^ ^]^

### Rheological Analysis

The study of the material's rheological characteristics was performed using a rheometer (AR2000, TA Instruments, USA), employing a parallel plate setup with disk specimens measuring 1.0 mm in thickness and 25 mm in diameter. Temperature ramps were set from 0 to 250 °C, with a rate of temperature increase of 3 °C min^−1^, while maintaining a strain amplitude at 1% and an angular frequency at 1 rad s^−1^. Frequency sweeps were conducted at a temperature of 75 °C in the range from 1 to 100 rad s^−1^ and 1% of strain amplitude. Then, the measured data was analyzed using the thermal analysis and rheology software (TRIOS, TA Instruments, USA).

### DSC

DSC analysis was performed using a thermal analysis system (DSC 3, Mettler‐Toledo International Inc., Switzerland). SMP pellets were dried in a vacuum oven (Vacucell EVO 55, MMM Medcenter Einrichtungen GmbH, Germany) at 80 °C for 12 h before analysis. Then, a pellet was cut into a small size, 10.5 mg weight, and sealed in a 40 µL standard aluminum crucible for the measurement. The DSC measurement involved 10 cycles of heating and cooling at a rate of 10 °C min^−1^, ranging from 0 to 60 °C (Figure S6, Supporting Information). Then, using DSC analysis software (STARe evaluation, Mettler‐Toledo International Inc., Switzerland), *T*_g_, *T*_start_, and *T*_end_ were calculated from cycles 1 to 9. Cycle 0 was not used in the calculation as it removes the thermal history of the sample,^[^ ^63^ ^]^ providing more accurate results.

### Preform Preparation

A custom molding procedure was developed to make a preform, a cylindrical macroscopic version of the final SMPF on a large scale, as shown in Figure 1. Dried pellets were put in a custom mold (Figure 1a) to manufacture an H150 x D40 mm cylinder shape preform. Steel rods were installed in the mold to create the hollow channels in the preforms. Both steel rods and the inside surface of the mold were coated with Polytetrafluoroethylene (AFC2201, AFT Fluorotec Ltd., UK) with a 50–100 µm thickness. This coating allows easy removal of the preform from the mold. Molds for each demonstrator were prepared with different numbers of rods; 6 sides and a single center channel for the catheter demonstrator, a single center channel for the neural interface, and eight side channels for the cochlear demonstrator were used. A minimum of 160 g of SMP pellets were filled into the mold in each molding, and the mold was heated in the vacuum oven for 24 h at 215 °C while applying a weight of 60 kg to the SMP material. The mold was chilled at 10 °C for 2 h, keeping the weight. The SMP preform was then released from the mold.

### Fabrication of SMPFs

The preform was attached to the custom‐built draw tower, as shown in Figure 1b. During the heating period, three zones in the heating chamber were set to 140, 215, and 70 °C, respectively. During the drawing, the diameter of the drawn fiber was continuously measured using a laser scan micrometer (TLAser222, Laserlinc, Inc., USA). The down feed speed was set to 1 mm min^−1^, and by adjusting the capstan speed, fibers were drawn in diameters ranging from 0.6 to 2.8 mm. In addition to the SMPFs with seven lumens for the catheter demonstrator in Figure 1e, a custom‐designed wire feeding mechanism (Figure 1c) enabled the existing fiber drawing platform to continuously integrate 0.03 mm dimension Pt/Ir wires (PT02‐WR‐000103, Goodfellow Cambridge Ltd., UK) or 0.03 mm dimension stainless steel wires (The Crazy Wire Company Ltd., UK). These fibers incorporated a single Pt/Ir wire for the neural interface (a commonly used electrode material), as presented in Figure 1f, and eight stainless steel wires for the CI, as shown in Figure 1g.

### SMP Catheter—FEA

FEA was conducted using COMSOL to model the thermal dynamics of a Shape Memory Polymer Fiber (SMPF)‐based catheter. The catheter measured 70 cm in length with an outer diameter of 2.4 mm. It has a central lumen with a diameter of 1 mm and smaller peripheral lumens, each measuring 0.4 mm in diameter. For the simulation, specific material properties, including heat capacity and thermal conductivity, were incorporated where values were interpolated to reflect the conditions at varying temperatures. The heat capacity values used were 2.45 J/(kg·K) at 280 K, 2.6 J/(kg·K) at 305 K, 3.2 J/(kg·K) at 320 K, 3.1 J/(kg·K) at 360 K, and 3.0 J/(kg·K) at 370 K. The thermal conductivity values used were 0.2 W/(m·K) at 307.5 K and 0.225 W/(m·K) at 338 K, with the fiber material's density constant at 1250 kg m^−^^3^. Water flow rates varied between 28 and 50 mL min^−1^, with an inlet temperature set at 4.0 °C and the environmental temperature around the catheter consistently held at 37.0 °C. Temperature changes were meticulously assessed at eight strategic points along the catheter, spaced from the proximal to the distal end at 10 cm intervals, to analyze the impact of nonisothermal flow on the thermal behavior from the point of entry to the tip.

### Temperature Change Characterization

The experimental environment during characterizations was created to replicate the environment inside vascular structures. A bespoke water bath was used to provide a temperature‐controlled fluidic environment. The temperature inside the water bath was stabilized at 37.0 ± 0.5 °C, at which the SMP catheter is naturally soft due to its rubbery status. The coolant (water) was chilled to 4.0 ± 0.1 °C with the refrigerated circulator (RHC‐800, Cole‐Parmer Instrument Company, LLC, USA). Then, the 70 cm of the SMPF was merged into the water bath (37.0 ± 0.5 °C). The coolant's flow rate was adjusted from 28 to 50 mL min^−1^ via different pump output configurations. In each flow rate configuration, the coolant was injected for 1.5 min and repeated three times. The flow rate and temperature of the inlet coolant were monitored by a micro‐flow rate sensor (SLF3S‐1300F, Sensirion AG, Switzerland) mounted at the injection ports. The temperatures at the proximal and distal ends of the catheter were measured by the thermocouples (Type K Thermocouple 1/0.2 mm diameter, RS Components Ltd., UK) placed inside the working lumen. The entire setup of this hydraulic cooling system is illustrated in Figure S7 (Supporting Information).

### Assessing Stiffness Variance

The stiffness changes of the SMP catheter between stiff (cooled with 4.0 ± 0.1 °C coolant injected) and soft (natural at 37.3 ± 0.1 °C) statues were evaluated by conducting a flexural rigidity experiment at the catheter tip (50 mm length). A flow rate of 38 mL min^−1^ was selected, referring to Figure 3e, to cool the SMPF below *T*_start_ while minimizing the overall coolant injected into the body. The stiffness was tested by vertically pulling the catheter tip (50 mm) with a displacement of 10 mm three times. The overall vertical force applied was measured by a high‐precision force/torque sensor (Nano43, ATI Industrial Automation, USA). This process was repeated three times. The results were evaluated by assessing the total force (mN) applied for each unit (mm) of tip deflection. Notably, measurement for the catheter with coolant injection was performed after the catheter was fully cooled (>30 s), referring to Figure 3b. The experimental setups and theoretical details are explained in Figures S8 and S9 (Supporting Information).

### Phantom Study

Vasculature 1:1 scale abdominal soft silicon phantom with left tortuous iliac and celiac trunc (A‐S‐N‐004+, Elastrat, Switzerland) (Figure S10, Supporting Information) was used for this phantom study. A water bath (TR‐1A, AS ONE Corporation, Japan) was used to increase the water temperature (environment temperature) to 37.0 ± 0.4 °C, and the pump (PMC‐121B7B1, Sanso Electric Co., Japan) was used to circulate the water continuously through the phantom. The circulating warm fluid was dyed with red ink (T10, C. Josef Lamy GmbH, Germany) to visually represent circulatory dynamics resembling that of blood flow. The catheter accessed the phantom via a unidirectional valve, representing the catheter introducer in endovascular interventions. Then, the coolant (water) chilled to 4.0 ± 0.1 °C with the refrigerated circulator (RHC‐800, Cole‐Parmer Instrument Company, LLC, USA) was flown through the SMPF‐based catheter to adjust the stiffness.

### Neural Interface—Stiffness Measurement

A flexural rigidity experiment was conducted at the fiber tip (10 mm in length, 0.3 mm in diameter) to measure the stiffness of the SMPF‐based neural interface when it is stiff (room temperature at 25 ± 1 °C) and soft (natural at 37.3 ± 0.1 °C). Vertical displacements of 2 mm were performed three times, and force change was recorded using a high‐precision force/torque sensor (Nano43, ATI Industrial Automation, USA). Stiffness values were calculated based on the total force (mN) and each unit (mm) of tip displacement.

### Electrochemical Impedance Spectrum

An SMPF with a Pt/Ir electrode was used as a neural interface. The electrodes in a 2 cm‐long SMPF to the copper wire with conductive silver paint were manually connected. Then, a potentiostat (Interface 1010E, Gamry Instruments, USA) was utilized to perform the impedance measurements with an electrolyte of 1x phosphate‐buffered saline (PBS, Thermo Fisher Scientific Inc., USA). A three‐electrode measurement setting was chosen with the fiber's electrode as the working electrode, a Silver/Silver Chloride (Ag/AgCl) electrode (Cole‐Parmer Instrument Company, LLC, USA) as the reference electrode, and a Pt wire (Baioanalytical Systems, Inc., USA) as the counter electrode. With an applied AC voltage of 10 mV with a frequency ranging from 100 Hz to 10 kHz, the impedance measurement results were recorded at least three times per sample.

### Neural Interface Assembly

The SMPF was first electrically connected to a copper wire. Then, the connected copper wire was soldered to pin connectors (Sullins Connector Solutions, Inc., USA), while the ground wire (Stainless steel wire, GoodFellow Cambridge Ltd., UK) was soldered to its neighboring pin. Then, 5‐min epoxy (Devcon, ITW Performance Polymers, USA) was applied to secure these wires and electrically insulate the exposed conducting parts.

### Surgical Procedures

All animal procedures were approved by the Virginia Tech Institutional Animal Care and Use Committee (IACUC protocol number: 20–200) and Institutional Biosafety Committee and carried out in accordance with the National Institutes of Health Guide for the Care and Use of Laboratory Animals. Male C57BL/6J mice (aged 7–9 weeks, weight of 25 ± 2 g, Jackson Laboratory) were set up on a stereotaxic apparatus (David Kopf Instruments, USA) and induced 1–5% isoflurane during the whole procedure (induction via chamber and nosecone) for anesthesia. Before surgery, the animal was subcutaneously administered with buprenorphine (0.05–0.1 mg kg^−1^) with a dose of carprofen (5 mg kg^−1^) and Baytril (5 mg kg^−1^). A dental drill was used to expose the scalp for fiber implantation to create a small craniotomy where the fiber's electrode was further inserted by a micropositioner according to the brain atlas. The coordinates used here is −2.0 mm anteroposterior, −1.5 mm mediolateral, and −1.9 mm dorsoventral (relative to bregma). Another small craniotomy was made for a miniature screw (J.I. Morris Company, USA) as a ground where the stainless steel wire was further soldered to and followed by the fixation process by Metabond covering all the exposed skull area. Additional dental cement was applied to secure the implantation and make sure the implanted device was fully supported by the skull. A heating pad was provided during the implantation surgery as well as the recovery period till the animal regained consciousness.

### In Vivo Electrophysiology

Both acute and chronic recordings were performed. During the electrophysiology recording procedure, the pin connector was connected to a 32‐channel Neurophysiology System (Tucker‐Davis Technologies, USA). The sampling frequency of the recording was at ≈50 kHz.

### Statistical Analysis

The raw electrophysiological signals from the hippocampus of wild‐type mice brains (*n* = 5) were sampled at 48828 Hz. The recorded neural activities were bandpass‐filtered from 0.3 to 300 Hz for extraction of local field potentials and 300–5000 Hz for detection of single‐unit activity. To extract and sort the action potentials into clusters, we used a voltage threshold to detect the spikes with a limitation on the refractory period of 1 ms. The autocorrelation of each cluster was visually inspected so as not to violate the refectory period. PCA was applied to the extracted action potential for spike sorting. K‐means clustering was used to perform clustering. L‐ratio (<0.05) and ID (>20) of the separated clusters were used to evaluate the quality of clustered data. The data is represented as mean ± standard deviation. Statistical analyses and figures were generated using custom scripts developed in MATLAB (The MathWorks Inc., USA).

### CI—SMPF Twisting Setup

The setup was designed with a Dynamixel motor (ROBOTIS Co., Ltd., Republic of Korea) used to twist the SMPFs around their axis. The setup features a horizontal rail to allow for samples of variable length and a rotationally static slider to hold the SMPF in place. A constant‐force spring of 0.1 N was used to allow for constant tension on the 0.6 mm (± 0.05) and 1 mm (± 0.05) diameter SMPF during the twisting process while compensating for the slight shortening (up to 0.6 ± 0.1 mm in 50 mm length) of the twisted fibers. Each steel wire can withstand a minimum of 0.35 N, which is sufficient to prevent breakage during twisting. Once twisted, SMPFs were heated to 80.0 ± 5.0 °C for at least 2 min for the relaxation of SMPFs. The setup features a modular hot‐air distributor, as shown in Figure S12 (Supporting Information). The system is designed to route the heated air to a central point at its opening and provide even heating of the SMPF while it is mounted to the twisting setup. Temperatures were stable after 180 s of preheating for a 2‐segment heating system (50–70 mm SMPFs). Fluctuations of less than ±5.0 °C were measured at different points along the SMPF, indicating a homogeneous heat distribution during relaxation using the setup. Following the twisting process, the wires were manually pulled to test the integrity of the implant. This test did not result in any detachment of the wires.

### Simulations and Measurement to Evaluate the Effect of Twisting on Stiffness

FEA was performed in Comsol Multiphysics (Comsol AB, Sweden) (Figure S11, Supporting Information). The simulation involved a parametric sweep for an SMPF of 1 ± 0.05 mm diameter and 10 mm length with increasingly twisted geometry under 0.1 N load. A forced displacement between 1 and 4 mm in increments of 1.0 mm was applied to one end of the SMPF, and the resulting forces were evaluated numerically. The simulation results were validated experimentally using a microforce sensor (FT‐S100'000, FemtoTools AG, Switzerland). SMPFs with straight and helical electrodes having the same parameters as the simulated SMPFs were deflected perpendicularly at their tip, and the resulting forces were measured. The experiment was repeated at room temperature (25 ± 1 °C) as well as in heated air (39 ± 2 °C) to demonstrate the SMP softening effect above body temperature.

### Molding Procedure for SMPF

A flat mold was designed to match the CT imaging‐based average dimensions of human cochleae defined by R. Clark et al.^[^ ^64^ ^]^ As the commercial CI's diameter is ≈0.3–0.8 mm, the average dimensions of human cochleae were scaled (1.3x) for compatibility with the 0.6 and 1.0 mm diameter SMPFs with a 60 mm length. The molds were 3D printed (Form 3, Formlabs Inc., USA) using a resin (Formlabs White Resin V4, Formlabs Inc., USA). The SMPF was heated to 140.0 ± 6.0 °C and manually placed into the mold. Then, it was cooled for 1 h and removed from the mold.

### Design of Cochlear Phantom

A 3D model (Figure S13, Supporting Information) was created using a parametric model of the average dimensions of human cochleae.^[^ ^64^ ^]^ Then, it was used as a reference for developing a cochlear phantom. This cochlear phantom setup was designed to replicate the insertion of an SMPF through the round window at 39.0 ± 0.2 °C, using a cooled copper tube through which the straightened SMPF is inserted into the phantom. The copper tube is cooled by a surrounding water container that is kept at 4.0 ± 0.1 °C, resulting in an internal temperature of 10–15 °C.

### Animal Testing Disclosure

This study involved the use of mice, with detailed ethical considerations and testing protocols extensively outlined in the Surgical Procedures in 4.3. Experimental setup section. All animal testing procedures were approved by the Virginia Tech Institutional Animal Care and Use Committee (IACUC protocol number: 20–200) and Institutional Biosafety Committee and carried out by the National Institutes of Health Guide for the Care and Use of Laboratory Animals. Efforts were made to minimize animal suffering and ensure welfare by ethical best practices.

## Conflict of Interest

The patent US2022168946A1 is filed by IP2IPO INNOVATIONS LTD, published on 2 June 2022, with authors B.T., M.E.M.K.A., and G.Z.Y. on the patent. The authors declare that they have no other competing interests.

## Supporting information



Supporting Information

Supplemental Video 1

Supplemental Video 2

## Data Availability

The data that support the findings of this study are available in the supplementary material of this article.
