# Supplementary material for: Thermally Drawn Shape and Stiffness Programmable Fibers for Medical Devices
Source: Adv Healthc Mater. 2024 Dec 31;14(10):2403235. doi: 10.1002/adhm.202403235 (PMC12004436; doi:10.1002/adhm.202403235)
Supplement: Supplementary file 1 — Supporting Information [file ADHM-14-0-s002.docx]

Supporting Information

**Thermally Drawn Shape and Stiffness Programmable Fibers for Medical Devices**

*Jiwoo Choi,^+^ Qindong Zheng,^+^ Mohamed E. M. K. Abdelaziz,^+^ Thomas Dysli, Daniel Bautista-Salinas, Andreas Leber, Shan Jiang, Jianan Zhang, Ali Anil Demircali, Jinshi Zhao, Yue Liu, Nick W.F. Linton, Fabien Sorin, Xiaoting Jia, Eric M. Yeatman, Guang-Zhong Yang and Burak Temelkuran^*^*


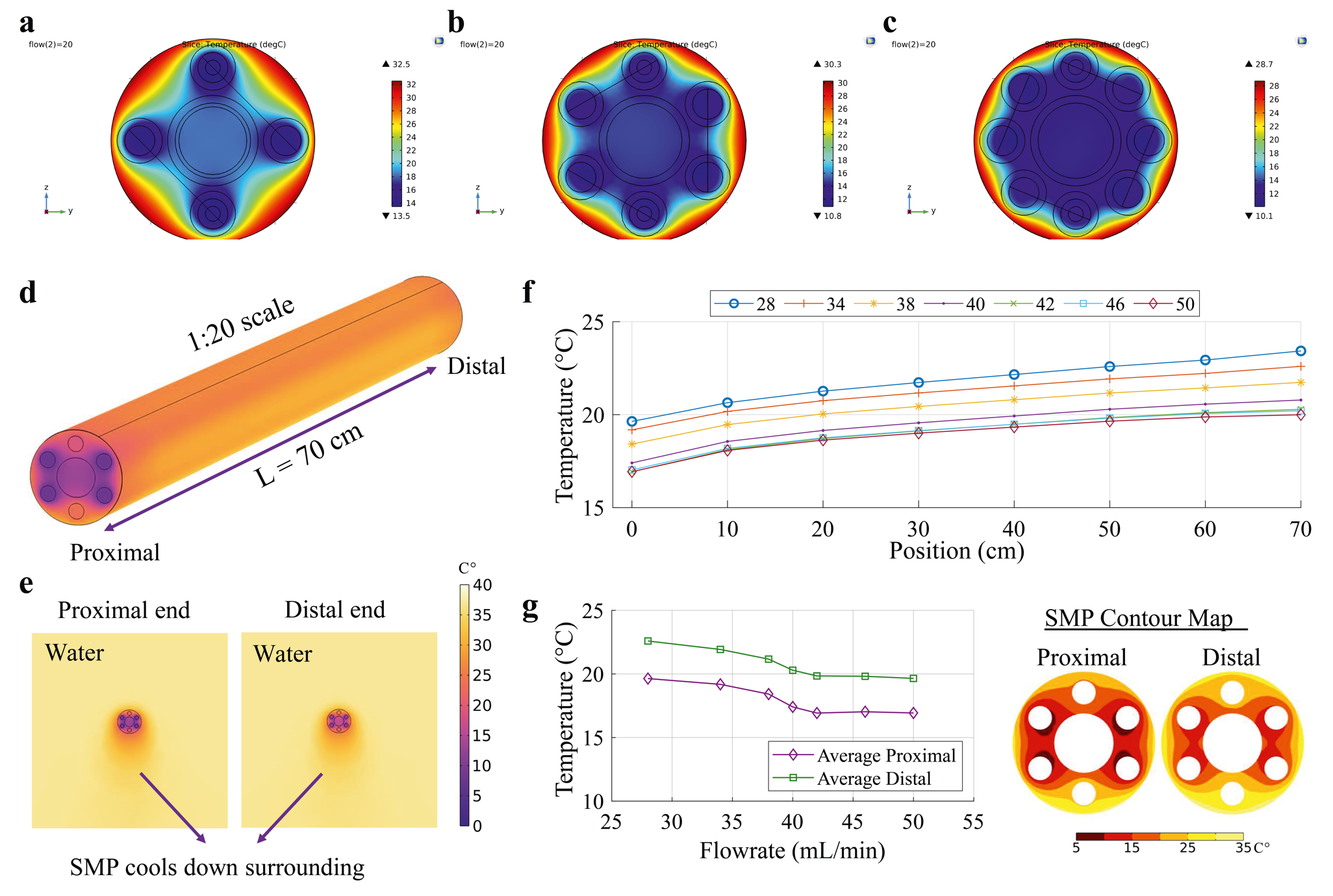


**Figure S1** Heat transfer analysis considering non-isothermal flow in a SMPF-based catheter device. (a-c) Heat transfer analysis in 3 different structural design with 4, 6, and 8 channels. (d) The isometric view of the SMP fibre with a 1:20 scale representation (L = 70 cm) is color-coded to indicate temperature distribution from the proximal to the distal end. (e) The cross-sectional views at the proximal and distal ends in the water that proves that SMP cools down its surroundings with water flow (40 mL/min is used for illustration). (f) The average temperature profiles at different positions along the SMP’s length for various water flow rates (i.e., 28, 34, 38, 40, 42, 46, 50 mL/min), with each line representing a different flow rate. Cross-sections are correlated with the position specified (i.e., proximal to the distal end; 0 to 70cm with 10 cm interval) to demonstrate SMP cross-section temperature is rising from the proximal end to the distal end due to the nature of the non-isothermal flow (40 mL/min is used). (g) The average temperatures at the proximal and distal ends of the SMP as a function of flow rate, accompanied by a contour map showing the temperature distribution across cross-sections of the SMP at both ends.





**Figure S2** Comparison of multilumen SMPF, commercial catheters, and thermally drawn fiber made from polycarbonate and polyetherimide. The flexural rigidity of each commercial catheter’s tube and sheath was measured using a cantilever beam theory. Commercial catheters were measured in the dry condition, and SMPF was measured while it was in the heated water bath without cooling (37.3 ± 0.1 ℃) and with cooling (20.4 ± 0.2 ℃). Mean ± SD, *n* = 3.


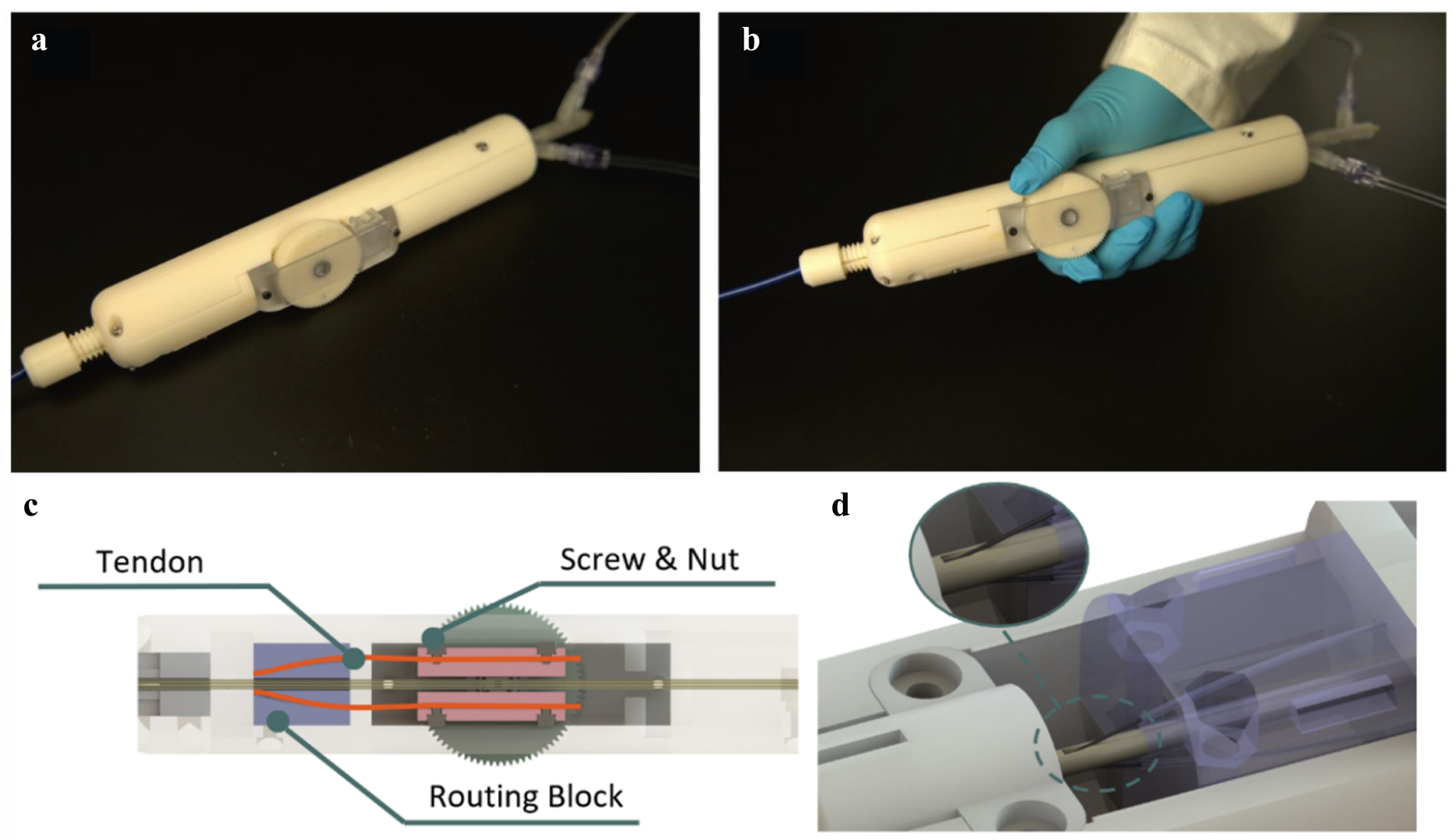
**Figure S3** A tendon-driven custom handle design (a) Image of the fabricated handle. (b) Ergonomic handling of the handle. (c) Internal design of the handle (d) Pulling wire integration with the manual handle.

**Pulling wire integration:** The cross-section of the SMP fiber for functionalization possesses two oppositely located pull-wire channels, allowing the transition of the pulling wires therein.  For the integration of the pulling wires, the wires are inserted into the corresponding channels via the pre-cut accesses and glued at the distal end. At the proximal end, the exposed wires are assembled with a bespoke manual handle, enabling the control of tendons. These integrated pulling wires allow a bi-directional tip deflection by manipulating the back-and-forth movement of the wires. Concerning the adaption of the variable stiffness property of the SMP catheter, the catheter shaft is required to remain stiff in the rubbery state so that only the catheter tip is soft and deflectable. Thus, a sheath with high stiffness is used to cover the catheter shaft, applying additional stiffness when the whole catheter shifts to a soft state.

**Steerability functionalization:** The catheter’s tip is operated via a tendon-based mechanism featuring two pulling wires (nitinol, 0.2 mm diameter) situated in lumens at (180°) angles within the fiber structure. These wires are manually threaded through the SMPF, and a stainless-steel tube (0.3 mm ID, 0.5 mm OD) was clamped on the tip and secured at the tip of the SMPF using a UV glue (Loctite, Loctite 3211, Germany). By securing the tendons at the SMPF’s distal end and pulling them at the proximal end, SMPFs are induced to bend in a controlled manner. This controlled bending, when combined with the bespoke catheter handle, provides manual control for steering. Details of the pulling wires integration and steering functionalization are shown in **Figure S3**.


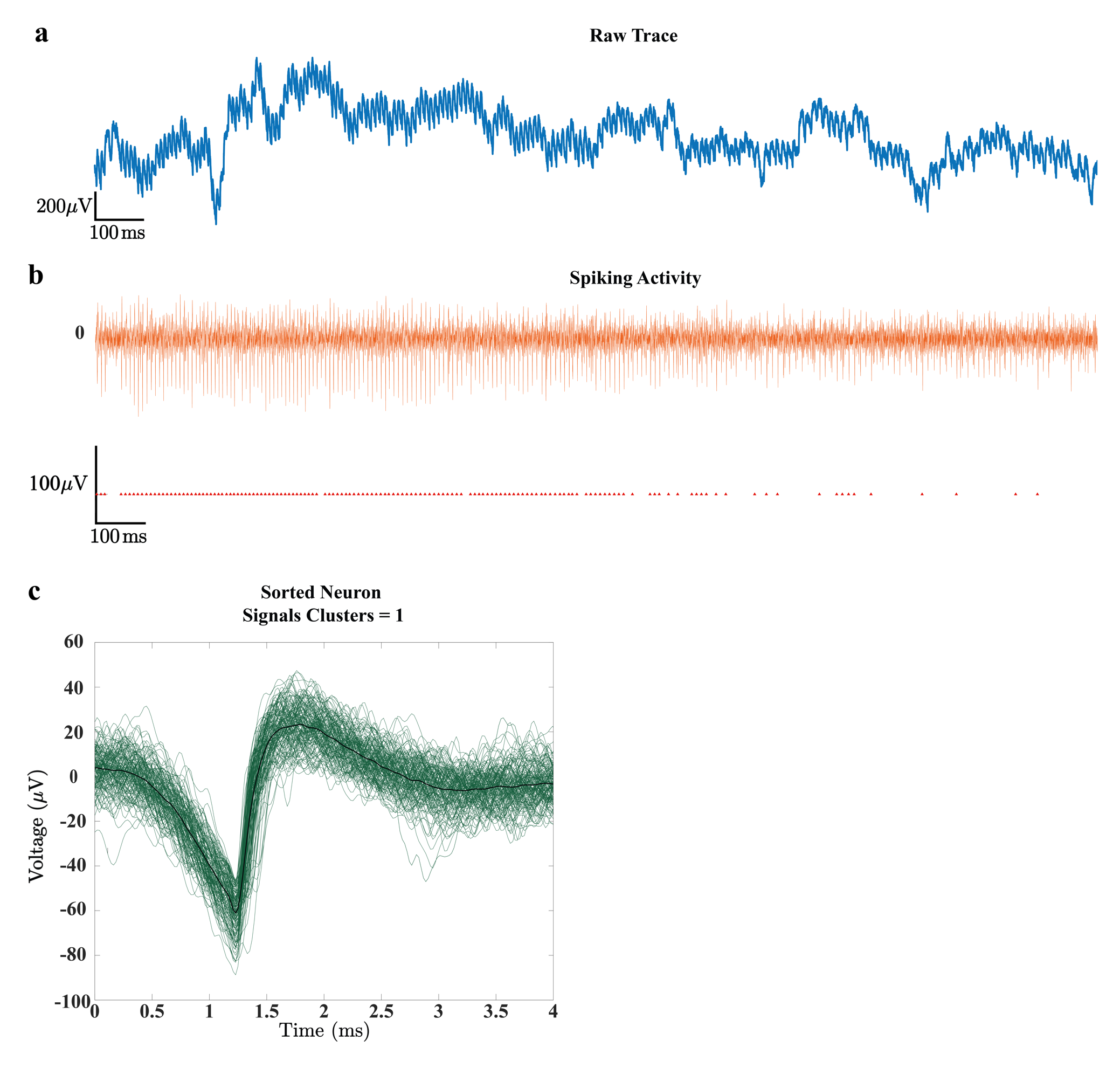


**Figure S4** SMPF-based neural interface’s measurement after 35 days post-implantation. (a) Raw neural signal (unfiltered). (b) High-frequency bandpass-filtered signal (300 to 5000 Hz, capturing single-unit activities). (c) Clustered neural signal. The electrophysiological recording procedure was repeated to assess the neural interface in chronic recording from the same five implanted mice 35 days after implantation. Solid line and shaded areas in the figure represent mean and standard deviation, respectively.


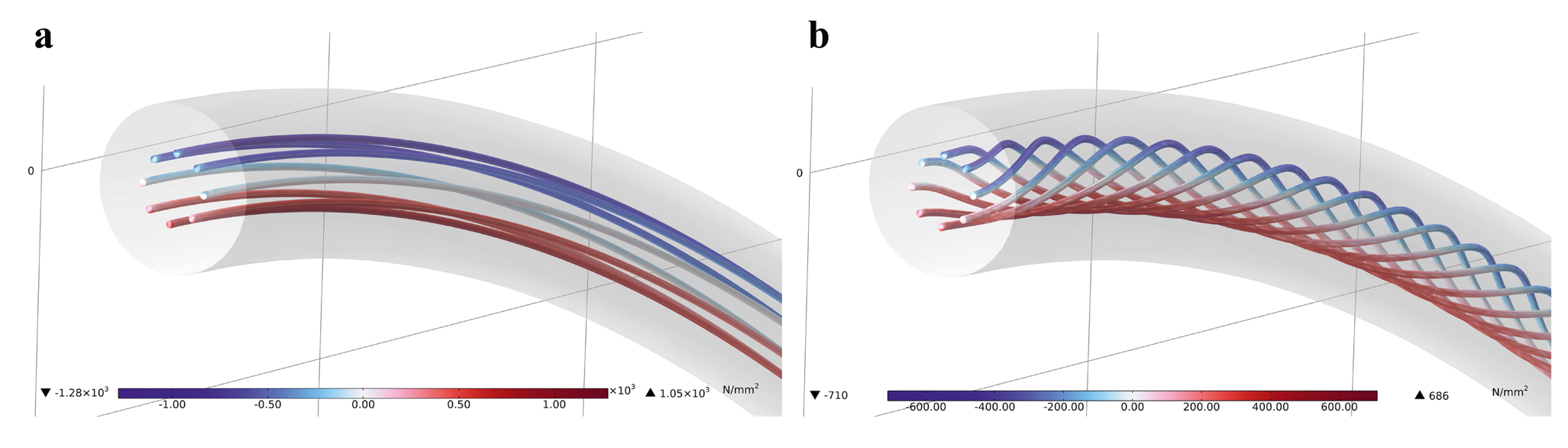


**Figure S5** Visualisation of tension and compression of the outer and inner wires. (a) The simulation of SMPF with straight wires. (b) The simulation of SMPF with 2.5 mm pitch helical wires.


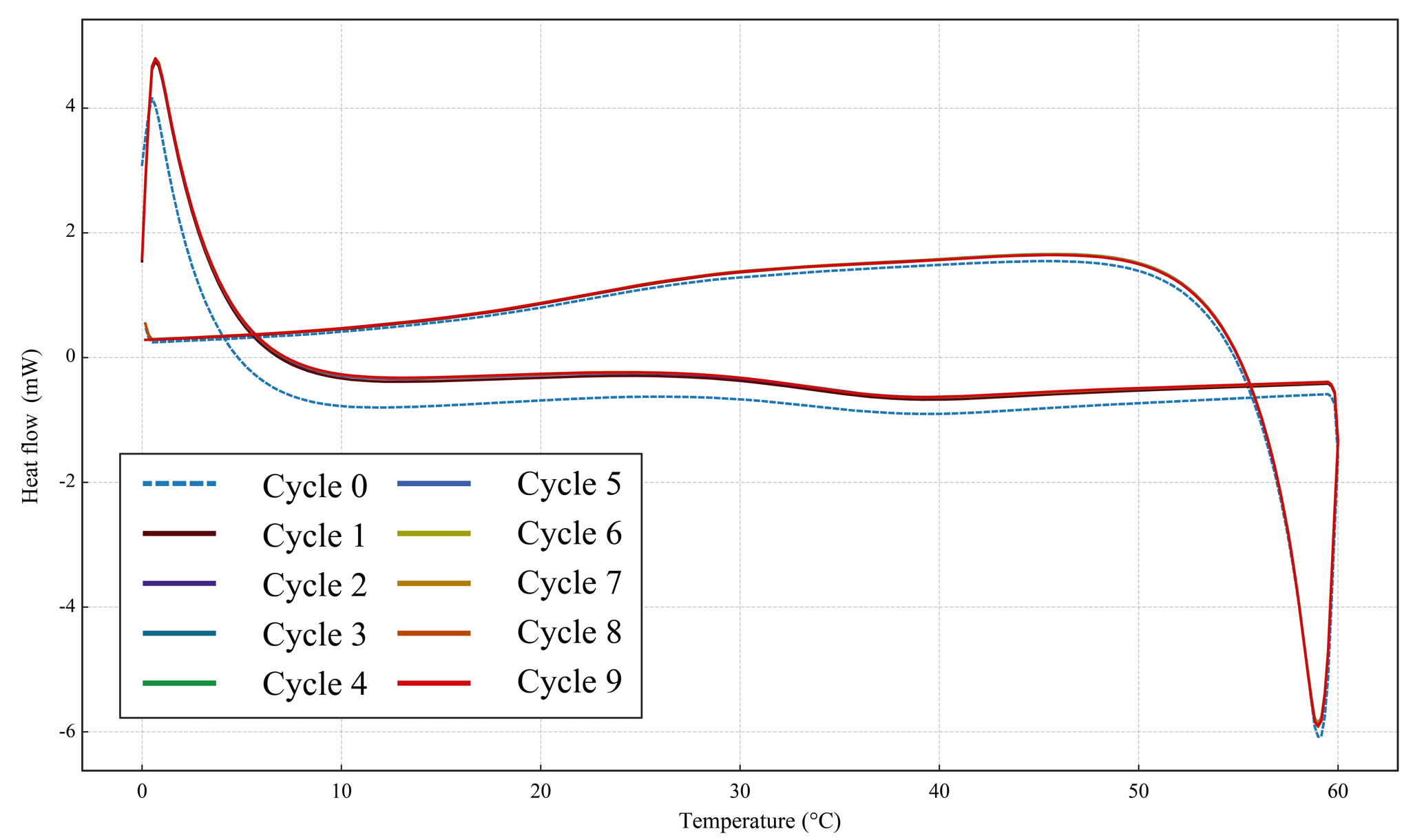


**Figure S6** Differential Scanning Calorimetry (DSC) analysis results. 10 cycles of 10 ºC/min heating and cooling rate in the range of 0 °C to 60 °C were used for measurement, with a sample size of 10.5 mg. *T_g_* was calculated from cycles 1 to 9 to improve the reliability of the measurement by removing any thermal history and erasing physical aging.^[1]^

*
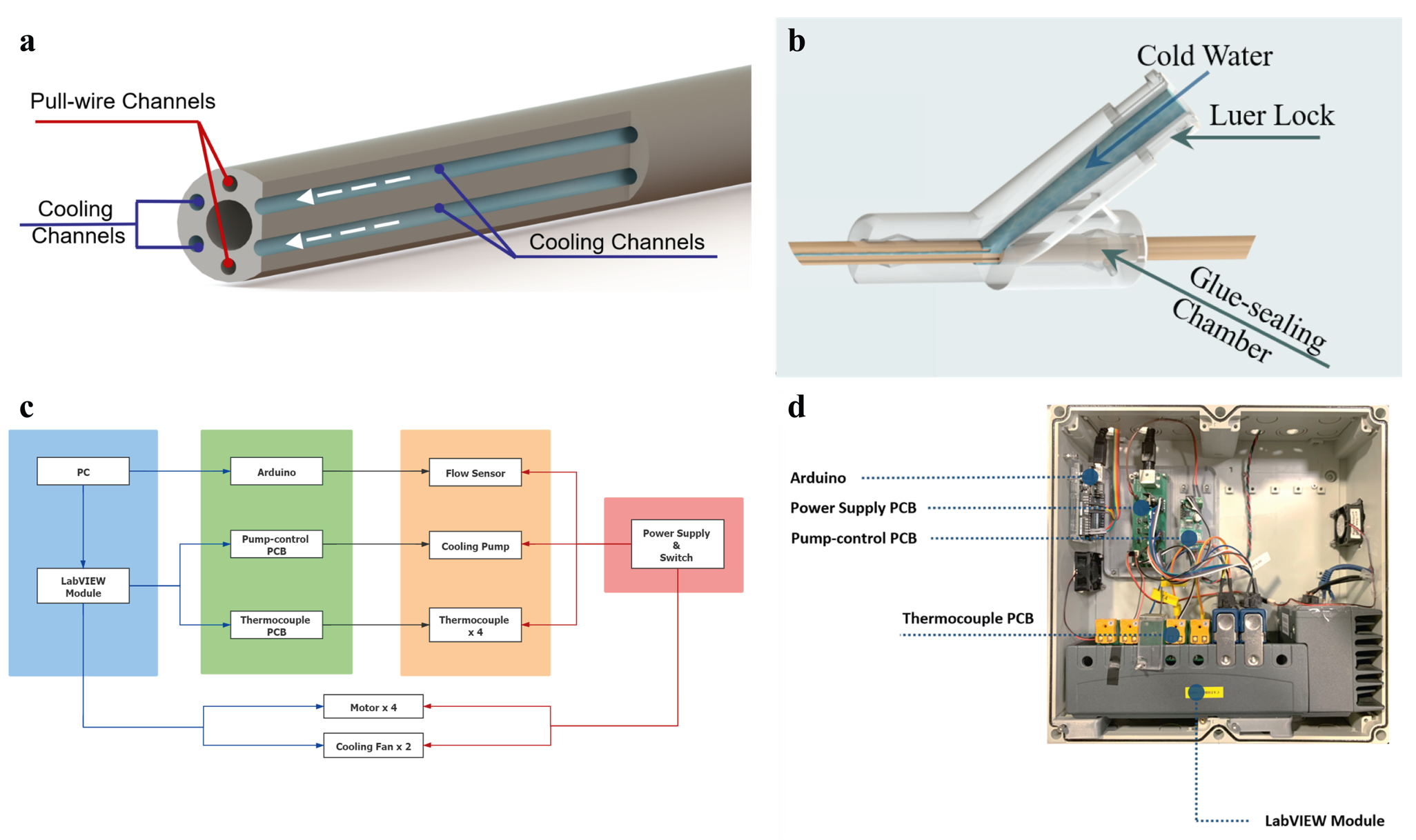
***Figure S7** Hydraulic cooling system. (a) Cooling at the catheter tip. (b) Fluid-Y-connector. (c) Schematic graph of the system. (d) Overview of the system composing the LABVIEW module and electronics.

**Cooling system:** The cooling system encompasses a control module, electronic module, flow rate sensor, pump, and cooler. Room temperature water is cooled down by a cooler (TR-1AR Thermal Robo, AS One Corporation, Japan) at 4 ± 1 ºC. The cooled water is then collected by a micro pump (MG1000, TCS Micropumps) and injected into the fluid channels (**Figure S6a**) via a bespoke connector named fluid-Y-connector in **Figure S6b**. The pump enables delivering consistent flow with a micro flow rate, offering an 8-bar maximum working pressure. The inlet port of the connector is sealed by a lure lock to prevent leakage.  **Figure S6c** shows the schematic flow chart and demonstration of the system. The hydraulic cooling system is controlled via LABVIEW interface in **Figure S6d**, which manipulates the power of pump output and monitors the data recorded. A liquid flow rate sensor (SLF3S-1300F, Sensirion) driven by Arduino is employed to measure and monitor the flow rate and temperature of the water input.

*
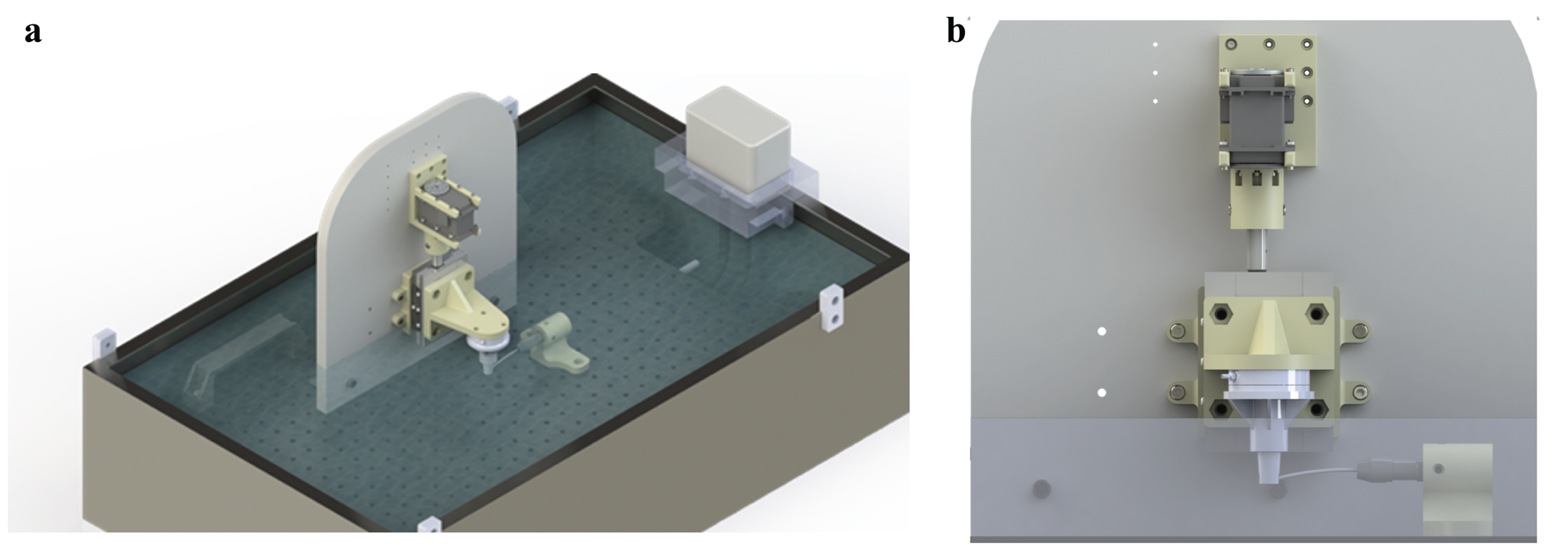
***Figure S8** Experimental setup for flexural rigidity measurements. (a) A temperature-controlled water bath experimental environment. This water bath is equipped with four water pumps underneath the water level, circulating the water and offering a consistent temperature along the bath. To measure the temperature of the catheter, one thermocouple was inserted into the central channel of the catheter and attached to the proximal end, and another thermocouple was attached to the distal end.  (b) The end of the catheter tip was fixed by a pin-vice, while its distal end was attached to a 3-D printed probe. A linear stage (1mm/rev) was controlled by a motor for the deflection of the catheter tip. A high-precision force/torque sensor (nano43, ATI Industrial Automation, USA) was attached to the probe, which was used for pulling the catheter tip. This force sensor allows the measurement of the vertical forces applied during tip deflection.

**Flow rate characterization:** mainly involves the measurement of volumetric flow rate (*Q*) within the circulation, by which flow velocity (*v*) and Reynolds number (*Re*) can be calculated accordingly. The purpose of this experiment is to measure the controllable range of flow rate and indicate the corresponding flow state, i.e., laminar, transition, or turbulent flow. The volumetric flow rate (*Q*) can be directly measured by the flow rate sensor (Sensirion SLF3S-1300F). The liquid from the outlet is collected and weighted by a high-precision scale to cross-validate the flow rate read by the flow sensor. By using the recorded flow rate, the flow velocity inside each fluidic channel can be calculated as:

$v=QA$ *(1)*

Where A is the cross-sectional area of each fluidic channel, which has an internal diameter (D) of 0.4 mm. The Reynolds number can be then evaluated as:

$Re=\frac{vD}{V_{cin}}$ *(2)*

*V_cin_* is the kinematic viscosity of the cooling water. *V_cin_* of water drops with the rising temperature. It is hypothesized that the cooling water reaches its maximum *Re* at the outlet of the channel. Thus, the temperature at the outlet is used to define *V_cin_*. Notably, *Re* smaller than 2100 is defined as laminar, *Re* between 2100 and 4000 is defined as transition, and *Re* larger than 4000 is defined as turbulent.

**Cooling efficiency characterization:** mainly involves the cooling performance of the system, i.e., temperature distribution and temperature drop time. Theoretically, the injected cold water is realizing its cooling function by creating a heat flux and dissipating heat from the SMP catheter. The form of heat transfer among blood, SMP catheter, and cold water is dominated by thermal conduction, as described in Supplementary **Figure S9**. A heat flux is generated that transfers heat along a temperature gradient from the blood vessel to the fluidic channel, resulting in a temperature gradient along the solid layer in between, i.e., the cross-section of the SMP catheter. It is noted that the temperature gradient along SMP can be quantified by using Fourier’s law:

$Q=-k\left( \nabla T \right)$ *(3)*

$\nabla T=T_{2}-T_{1}$ *(4)*

Let *T_2_* and *T_1_* be the temperature at the distal end (*T_distal_*) and the proximal end (*T_proximal_*), respectively. Heat transfer (*Q*) can then be calculated based on the conductivity (*k*) of SMP. The temperature gradient inside SMP can then be obtained.


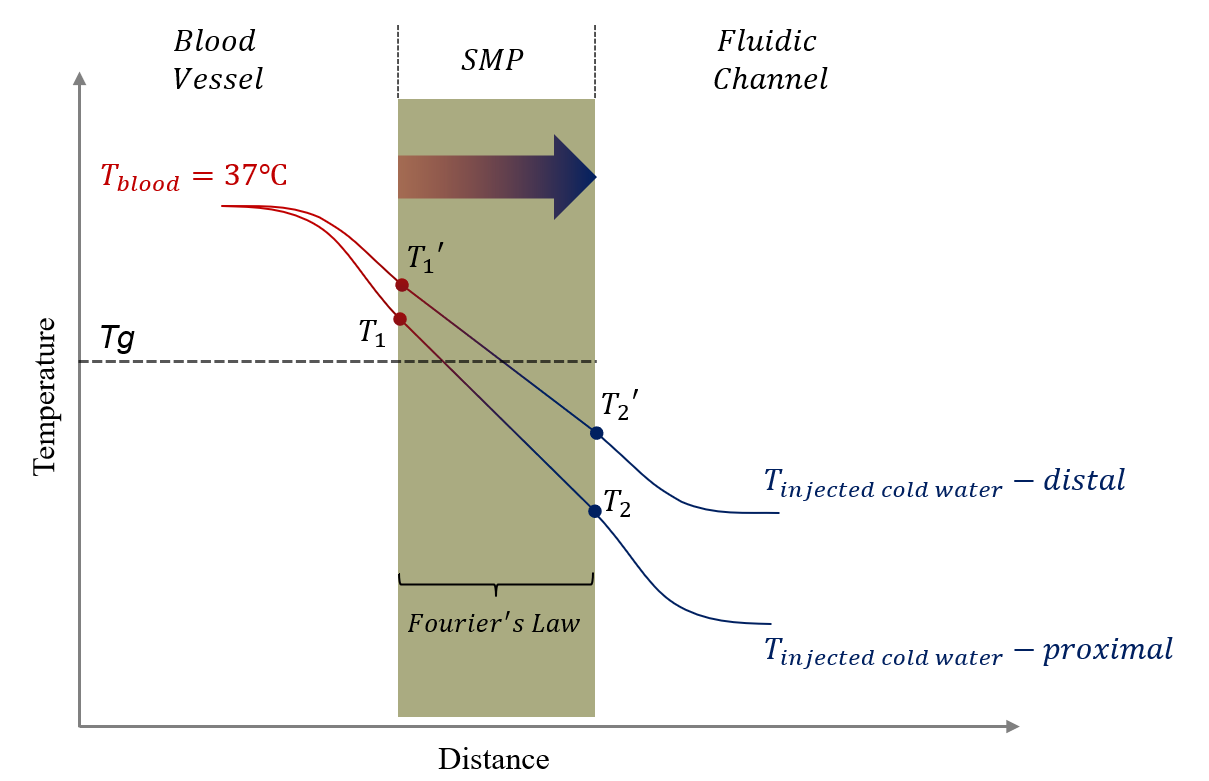
 **Figure S9** Conductive heat transfer among blood vessel, SMP, and fluidic channels.

Temperature drop time (*t_drop_*) is calculated to evaluate the response time of the cooling system. Notably, *t_drop_* is defined as the time for the temperature at the distal end to drop from 90% of the maximum to 110% of the minimum.

The cooling efficiency can be described as the total thermal energy of the SMP catheter that being dissipated by the cooling system. This can be evaluated by the overall heat increase of the injected cold water from the inlet to the outlet. In accordance with the theory of flow heat transfer, the cooling rate (*q.*) of the hydraulic cooling system can be calculated as:

$q.=m.C_{\rho}\Delta Tq.$ *(5)*

$m.=\rho Q$ *(6)*

Where *∆T* is the temperature difference between proximal and distal ends. Heat capacity C_p_ and ρ represent the specific heat capacity and density of water. m. is defined as the mass flow rate.

**Stiffness adjustability functionalization:** SMP composite with a *T_g_* of 35 ºC is selected in the thermally drawing fabrication process of the SMP catheter. The chosen *T_g_* allows the catheter to maintain a naturally soft status at physiological body temperatures. To harness variabilities in stiffness inherent to SMP, a hydraulic cooling system is developed to induce a transition of the SMP material into its glassy status by lowering the temperature of the catheter body below its *T_g_*. Coolant (4 ºC) is introduced into four symmetrically positioned fluid channels. This controlled infusion facilitates the reduction of temperature within the catheter body, thereby facilitating the transformation of the SMPF’s status from the rubbery phase to the glassy state.

**Flexural Rigidity Calculation:** The flexural rigidity of the beam was calculated using a cantilever beam theory:

$EI=\frac{{FL}^{3}}{3\delta}$ *(7)*

Where *E* represents Young's modulus, *I* is the second moment of area, *F* is the force applied at the tip, *L* is the length of the beam, and *δ* is the displacement at the tip. In this setup, the beam was subjected to a known force at its tip, and the resulting displacement was measured. By substituting the measured values of force, beam length, and displacement into the formula, the flexural rigidity, *EI*, was determined, providing a quantitative measure of the beam's stiffness in bending.

*
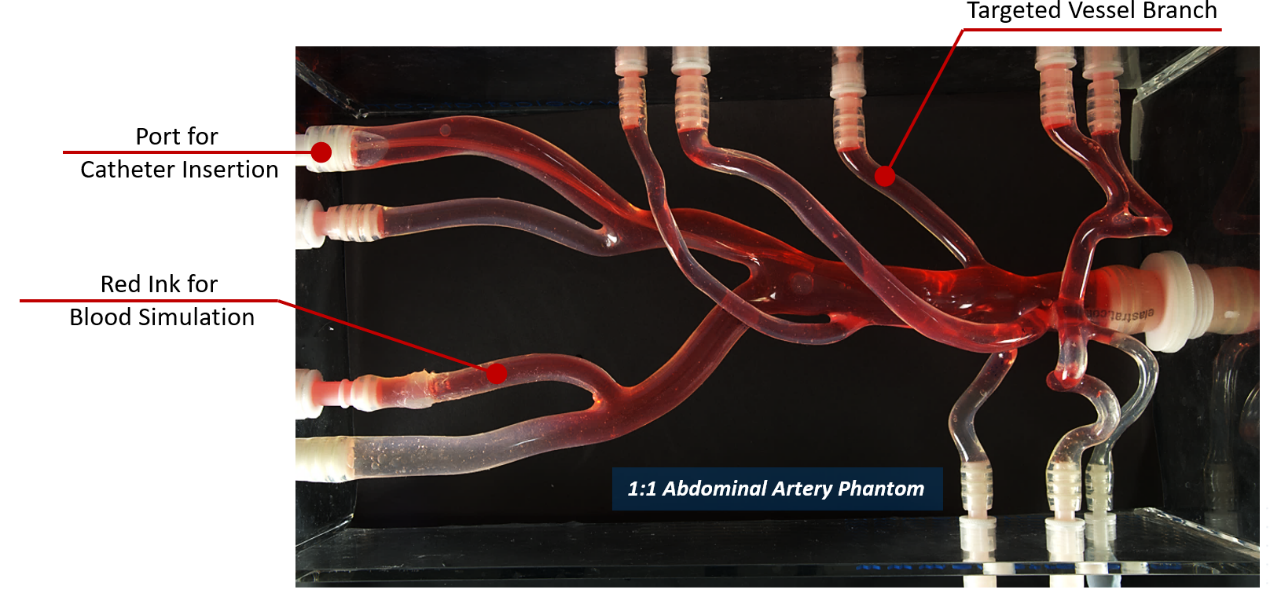
***Figure S10** Vasculature 1:1 scale abdominal soft silicon phantom with left tortuous iliac and celiac trunc (A-S-N-004+, Elastrat, Switzerland) phantom. The targeted vessel branch has a bending angle of over 120°.

**
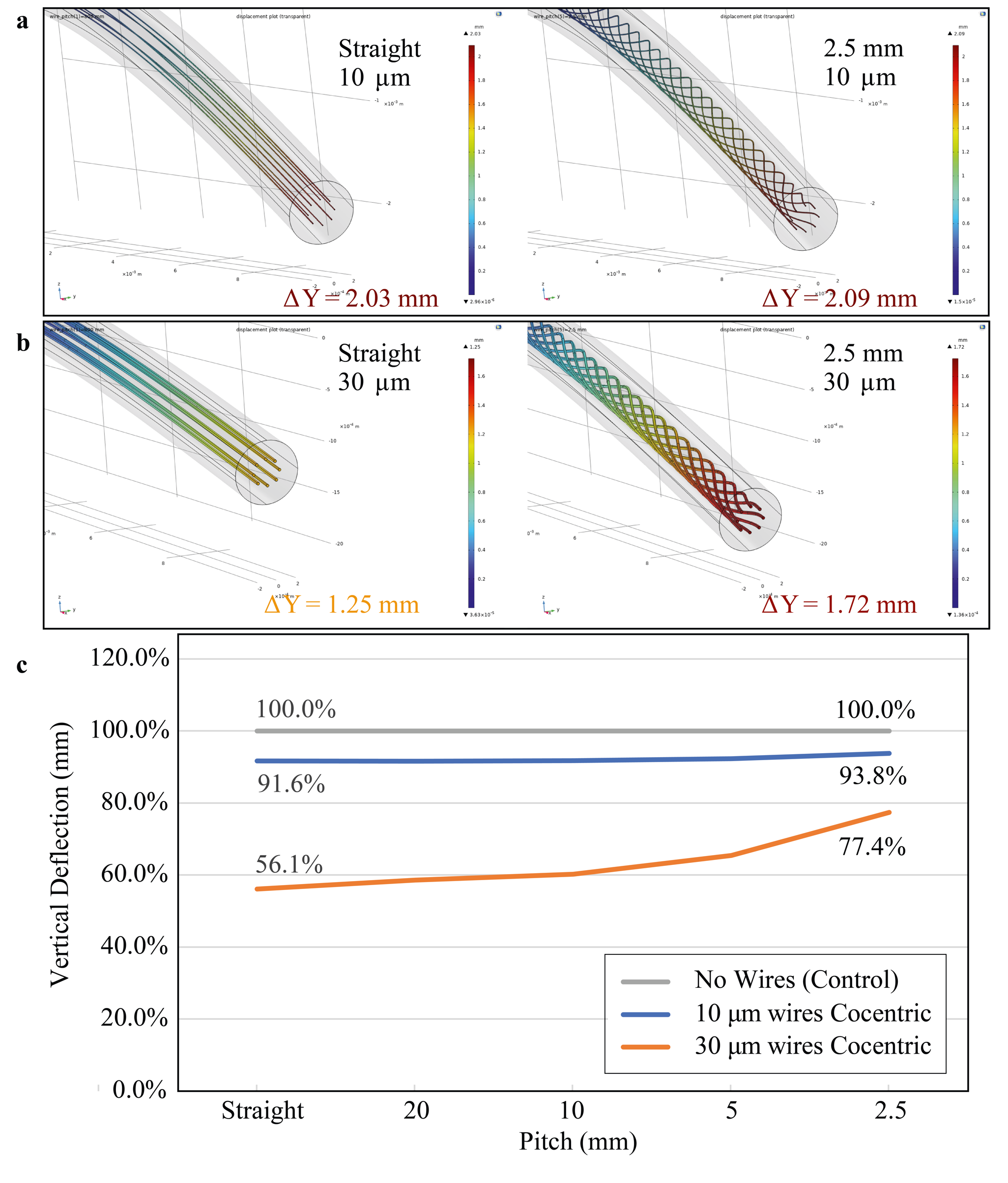
**

**Figure S11** Comsol simulation on the effect of the pitch on fiber stiffness. A 0.6mm SMPU fiber is simulated in COMSOL under 0.1 N load. (a) Simulation results of straight or twisted 10μm wires. (b) Simulation results of straight or twisted 30μm wires. (b) Simulation results in the percentage of deflection under the load.

*
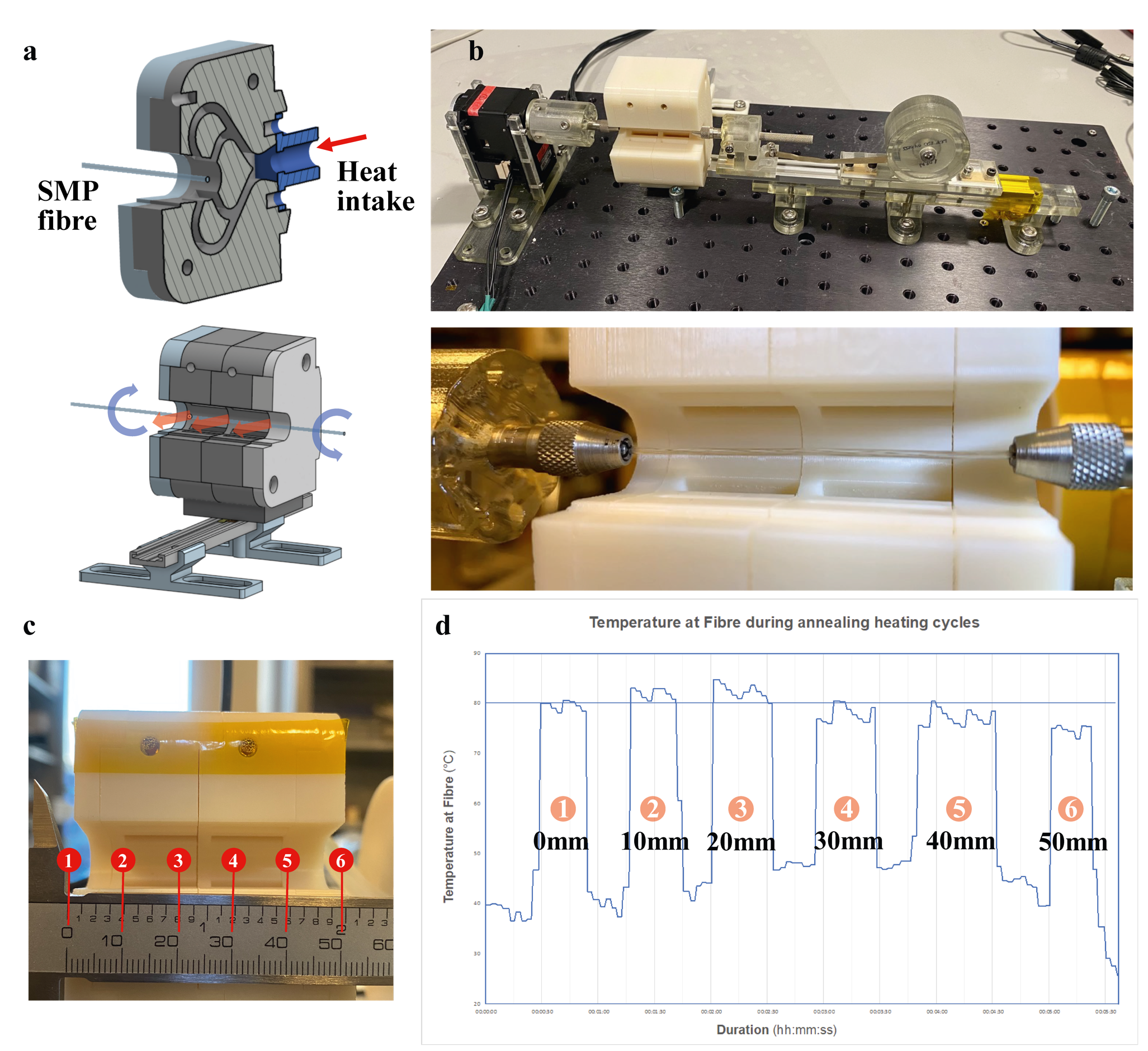
***Figure S12** Fiber heating and rotation setup at 80 ºC. (a) The heat was distributed to the fiber for 2 minutes during the winding. (b) The entire setup of fiber winding and heating setup. A heat gun was attached to this setup. (c) Distance of each measurement section in the 3D printed annealing module. (d) Measured temperature at the fiber surface during the annealing cycles.


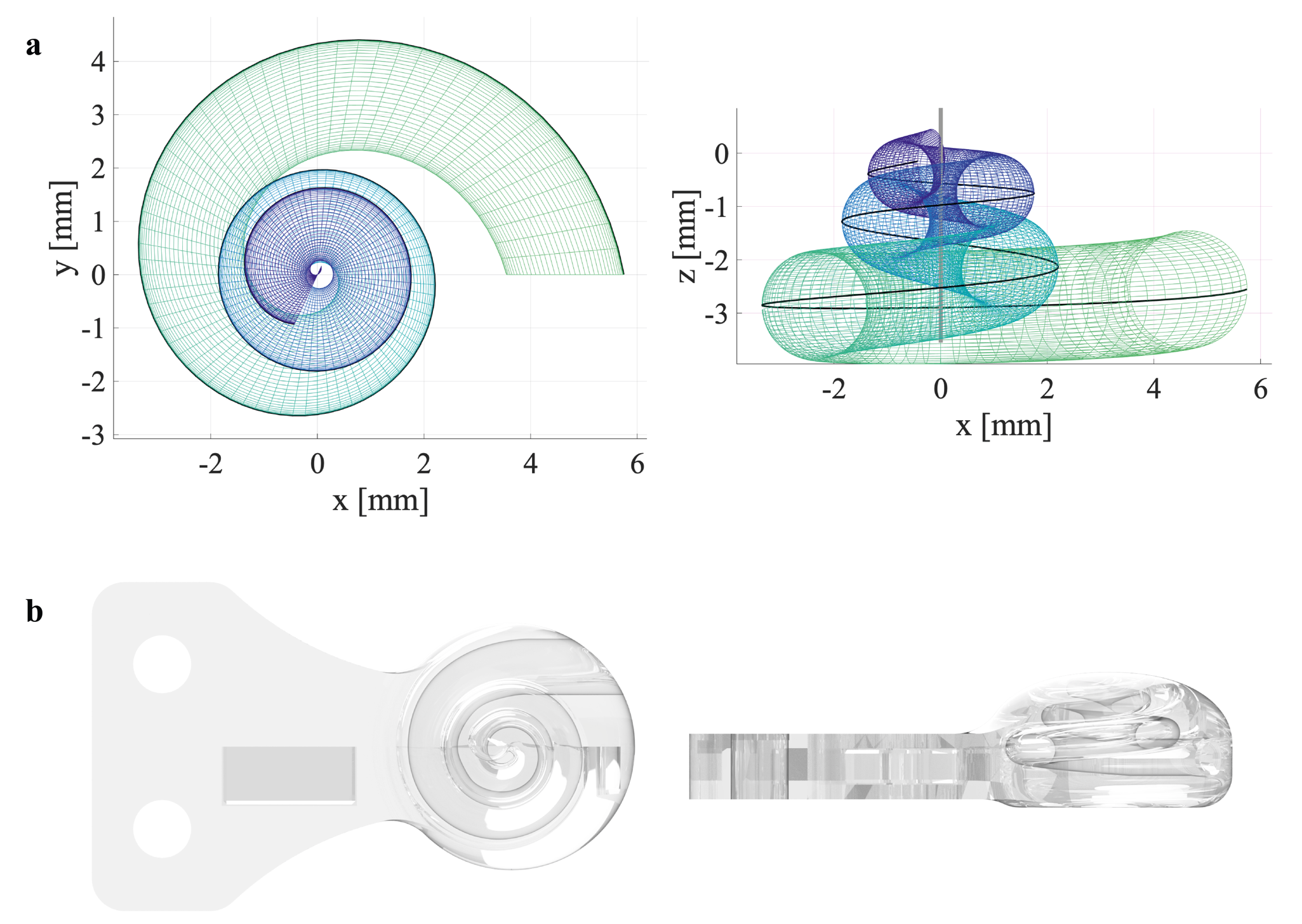
**Figure S13** 1:1.3 scaled phantom of the Scala Tympani (ST). (a) 3D mapping of cochelaer based on Clark et al.’s report.^[2]^ (b) 3D model of 12mm 1:1.3 scaled Scala Tympahni phantom.

**Table S1** Reported catheter-based intravascular flow Injection operations in clinics

| Catheter-based Intravascular Flow Injection Operation | Injection Fluid | Reported Flow Rate | Reported Fluid Volume (per operation) | Ref. |
| --- | --- | --- | --- | --- |
| Central Venous Access (Central Line Infusion) | 0.9% sodium chloride (saline), dextrose solution, parenteral nutrition, anesthetic, etc. | 90 mL/min with 16-gauge single CVC^1^ | 30 mL/kg (adult) in the first hour | ^[3]^ |
| Peripheral Intravenous Infusion |  | 350 mL/min with 14-gauge PIV^2^ |  |  |
| Contrast Injection | Contrast medium mixed with saline solution | Standard: 1.5-2.0 mL/sec  Rapid: 3.0-5.0 mL/sec | 150-200 mL | ^[4]^ |
| Saline Infusion through Ablation Catheter | 0.9% sodium chloride (saline) | Around 30 mL/min for RF energy > 30 watts | 1277±316 mL with commercial catheter TCAC^3^ | ^[5]^ |
| Cold Saline Infusion | 4 ºC 0.9% sodium chloride (saline) | 100 mL/min | 30 mL/kg (adult) | ^[6]^ |

^1^CVC: central venous catheter; ^2^PIV: peripheral intravenous catheter; ^3^TCAC: SmartTouch ThermoCool® ablation catheter

The direct intravascular injection of saline solution is a standard procedure in operation, such as saline infusion and injection of contrast agent mixed with saline solution. The flow rates used are generally higher than what we used in our experiment. As presented in the table, the saline solution is the most prevalent fluid for intravascular injection. The flow rate to patients up to 5 mL/sec (300 mL/min) was reported in rapid, dynamic, and discontinuous flow injections, such as contrast agent injection for fluoroscopy, CT scanning, MRI, etc. In continuous flow injection, a scenario comparable to our SMP catheter application is the injection of saline through an ablation catheter during invasive cardiac arrhythmia treatment, in which catheters are inserted percutaneously into the femoral vein. A flow rate of approximately 30 mL/min has been reported, with injected fluid volumes exceeding 1300 mL considered safe for patients over a typical operation duration. In extreme condition of cold intravenous saline infusion for inducing hypothermia, 4 ºC saline is administered at a rate of 100 mL/min, with a total volume of up to 2100 mL for a 70 kg patient (30 mL/kg). Such operations are proceeded peripherally, meaning that vessels with minor diameters are affected. Considering the size of our catheter, the vessels accessible are much larger ones and thereby more tolerable to stress increase. Functional ingredients, such as anesthetic and contrast agent, are usually mixed with 0.9% saline solution. Other alternatives include dextrose solution, parenteral nutrition, and blood, which can also be a candidate for coolant, but their applications in clinical scenarios are limited. Thus, saline solution will be the ideal biocompatible coolant for our application.

**Table S2** Existing stiffness adjustable catheters

|  | Cooling Method | Cooling Time | Stiffness Adjustability Ratio^1^ | Stiffness Adjustable Temperature Range | |
| --- | --- | --- | --- | --- | --- |
|  |  |  |  | *T_stiff_* (℃) | *T_soft_* (℃) |
| SMPF-based Catheter | Active | 8.4 s (at 38 mL/min) | 31-fold | 20 | 37 |
| SMP-based Magnetic Catheter^[7]^ | Active | 4.4 s (in air) | 66-fold | 25* | 80  marginal effect at over 60 |
| SMP-based Magnetic Catheter^[8]^ | Passive | 107 s (in air) | 21-fold | 23* | 80  marginal effect at over 60 |
| SMP-based Robotic Catheter^[9]^ | Passive | 87.8 s (in air) | Up to 38-fold | 23* | 80  marginal effect at over 60 |
| LMPA^2^ Magnetic Catheter^[10]^ | Passive | 80 s (in air)  20s (in water) | N/A | 24* (in air)  37 (in water) | 60 (in air)  41 (in water) |

*Namely, room temperature defined by each author

^1^Stiffness Adjustability Ratio: This parameter is defined as the ratio of flexural rigidity between the stiff and flexible states.

^2^LMPA: Low Melting Point Alloys

**References**

[1] J. M. Hutchinson, *J. Therm. Anal. Calorim.* **2009**, *98*, 579.

[2] J. R. Clark, F. M. Warren, J. J. Abbott, *J. Med. Devices.* **2011**, *5*, 014501.

[3] a)M. Malbrain, T. Langer, D. Annane, L. Gattinoni, P. Elbers, R. G. Hahn, I. De Laet, A. Minini, A. Wong, C. Ince, D. Muckart, M. Mythen, P. Caironi, N. Van Regenmortel, *Ann. Intensive Care* **2020**, *10*, 64; b)D. J. Berman, A. Schiavi, S. M. Frank, S. Duarte, D. A. Schwengel, C. R. Miller, *Transfusion* **2020**, *60*, 1410.

[4] a)B. R. Herts, C. M. O'Malley, S. L. Wirth, M. L. Lieber, B. Pohlman, *AJR Am. J. Roentgenol.* **2001**, *176*, 447; b)P. C. Sanelli, M. Deshmukh, I. Ougorets, R. Caiati, L. A. Heier, *AJR Am. J. Roentgenol.* **2004**, *183*, 1829.

[5] N. Chopra, A. K. Amin, A. Gupta, E. Y. Fu, A. J. Nichols, S. D. Nelson, J. M. Kleman, J. M. Kleman, G. A. Kidwell, S. R. Billakanty, *J. Atr. Fibrillation.* **2018**, *11*, 2093.

[6] I. M. Larsson, E. Wallin, S. Rubertsson, *Resuscitation* **2010**, *81*, 15.

[7] Y. Piskarev, Y. Sun, M. Righi, Q. Boehler, C. Chautems, C. Fischer, B. J. Nelson, J. Shintake, D. Floreano, *Adv. Sci. (Weinh.)* **2024**, *11*, 2305537.

[8] Y. Piskarev, J. Shintake, C. Chautems, J. Lussi, Q. Boehler, B. J. Nelson, D. Floreano, *Adv. Funct. Mater.* **2022**, *32*, 2107662.

[9] M. Mattmann, C. De Marco, F. Briatico, S. Tagliabue, A. Colusso, X. Z. Chen, J. Lussi, C. Chautems, S. Pané, B. Nelson, *Adv. Sci. (Weinh).* **2022**, *9*, 2103277.

[10] C. Chautems, A. Tonazzini, Q. Boehler, S. H. Jeong, D. Floreano, B. J. Nelson, *Adv. Intell. Syst.* **2019**, *2*, 1900086.
